# The testis-specific E3 ubiquitin ligase RNF133 is required for fecundity in mice

**DOI:** 10.1186/s12915-022-01368-2

**Published:** 2022-07-13

**Authors:** Kaori Nozawa, Yoshitaka Fujihara, Darius J. Devlin, Ricardo E. Deras, Katarzyna Kent, Irina V. Larina, Kohei Umezu, Zhifeng Yu, Courtney M. Sutton, Qiuji Ye, Laura K. Dean, Chihiro Emori, Masahito Ikawa, Thomas X. Garcia, Martin M. Matzuk

**Affiliations:** 1 Center for Drug Discovery, Baylor College of Medicine, Houston, TX 77030 USA; 2 Department of Pathology & Immunology, Baylor College of Medicine, Houston, TX 77030 USA; 3 Department of Bioscience and Genetics, National Cerebral and Cardiovascular Center, Suita, Osaka 564-8565 Japan; 4 Department of Molecular Physiology and Biophysics, Baylor College of Medicine, Houston, TX 77030 USA; 5 Department of Experimental Genome Research, Research Institute for Microbial Diseases, Osaka University, Suita, Osaka Japan

**Keywords:** Sperm defects, ERAD, Male infertility, Contraceptive, PROTACs

## Abstract

**Background:**

Ubiquitination is a post-translational modification required for a number of physiological functions regulating protein homeostasis, such as protein degradation. The endoplasmic reticulum (ER) quality control system recognizes and degrades proteins no longer needed in the ER through the ubiquitin–proteasome pathway. E2 and E3 enzymes containing a transmembrane domain have been shown to function in ER quality control. The ER transmembrane protein UBE2J1 is a E2 ubiquitin-conjugating enzyme reported to be essential for spermiogenesis at the elongating spermatid stage. Spermatids from *Ube2j1* KO male mice are believed to have defects in the dislocation step of ER quality control. However, associated E3 ubiquitin-protein ligases that function during spermatogenesis remain unknown.

**Results:**

We identified four evolutionarily conserved testis-specific E3 ubiquitin-protein ligases [RING finger protein 133 (*Rnf133*); RING finger protein 148 (*Rnf148*); RING finger protein 151 (*Rnf151*); and Zinc finger SWIM-type containing 2 (*Zswim2*)]. Using the CRISPR/Cas9 system, we generated and analyzed the fertility of mutant mice with null alleles for each of these E3-encoding genes, as well as double and triple knockout (KO) mice. Male fertility, male reproductive organ, and sperm-associated parameters were analyzed in detail. Fecundity remained largely unaffected in *Rnf148*, *Rnf151*, and *Zswim2* KO males; however, *Rnf133* KO males displayed severe subfertility. Additionally, *Rnf133* KO sperm exhibited abnormal morphology and reduced motility. Ultrastructural analysis demonstrated that cytoplasmic droplets were retained in *Rnf133* KO spermatozoa. Although *Rnf133* and *Rnf148* encode paralogous genes that are chromosomally linked and encode putative ER transmembrane E3 ubiquitin-protein ligases based on their protein structures, there was limited functional redundancy of these proteins. In addition, we identified UBE2J1 as an E2 ubiquitin-conjugating protein that interacts with RNF133.

**Conclusions:**

Our studies reveal that RNF133 is a testis-expressed E3 ubiquitin-protein ligase that plays a critical role for sperm function during spermiogenesis. Based on the presence of a transmembrane domain in RNF133 and its interaction with the ER containing E2 protein UBE2J1, we hypothesize that these ubiquitin-regulatory proteins function together in ER quality control during spermatogenesis.

**Supplementary Information:**

The online version contains supplementary material available at 10.1186/s12915-022-01368-2.

## Background

Ubiquitination is a post-translational modification of proteins that is required for many cellular processes, including protein degradation by the proteasome, and requires the sequential action of three enzymes called E1 (ubiquitin-activating enzyme), E2 (ubiquitin-conjugating enzyme), and E3 (ubiquitin ligase) [1, 2]. While there is a single E1 enzyme and ~ 40 E2 enzymes, there are over 600 E3 enzymes in humans, with the RING (Really Interesting Novel Gene) family of E3 ubiquitin ligases being the most abundant [1]. The RING E3 ubiquitin ligases recognize specific substrate proteins and interact with the ubiquitin-charged E2 enzymes to transfer the ubiquitin to the substrate [3]. Recently, this pathway has been utilized to target physiologically relevant proteins for degradation using PROteolysis TArgeting Chimeras (PROTACs) in which bifunctional molecules are synthesized to bind to both an E3 ubiquitin ligase and a target protein for specific degradation of a target [4, 5]. A significant advantage of this approach is that the target-binding molecule does not necessarily need to be a functional inhibitor; only the affinity for both the target and E3-ligase complex is necessary for proteolysis. As many potential targets for male contraception lack enzymatically active sites, where traditional inhibitors could act, PROTACs are an approach well-suited for the development of novel non-hormonal male contraceptives to target these non-enzymes.

Endoplasmic reticulum (ER)-associated degradation (ERAD), a central component of the secretory pathway in all eukaryotic cells, directs misfolded ER proteins for ubiquitination [6]. Because the ubiquitin–proteasome system is in the cytoplasm, there must be a mechanism to transport these misfolded proteins to the cytoplasm [7]. ER membrane-anchored RING finger E3 ubiquitin ligases are the major mediators of ubiquitination of these misfolded proteins during ERAD. Over 25 human transmembrane E3 proteins are predicted to be ER-localized, but the function of most of these proteins remains unknown. In a recent study, Fenech et al. [8] mapped over 450 protein–protein interactions for 21 transfected ER E3s including RNF148. Based on our analysis, RNF133 and RNF148 are predicted to be ER transmembrane paralogs and are chromosomally linked. Our knowledge of the roles of RNF133 and RNF148 and the ubiquitin–proteasome system in ER quality control and ERAD during spermatogenesis are limited. Studies conducted by Koenig et al. [9] revealed that the E2 ubiquitin-conjugating enzyme, UBE2J1, is required for spermiogenesis at the elongating spermatid stage. UBE2J1, like RNF133 and RNF148, is an ER transmembrane protein, and defects in the *Ube2j1* KO are believed to be secondary to a defect in the dislocation step of ER quality control [10]. Electron microscopy (EM) analysis of *Ube2j1* KO sperm reveals excess residual cytoplasm in the head and midpiece of the sperm [9].

Limited functional data is available for the ubiquitin–proteasome pathway in the male reproductive tract [11]. Using bioinformatics approaches, we uncovered several testis-specific E3 ubiquitin-protein ligases in mice and humans: RNF133, RNF148, RNF151, and ZSWIM2. We hypothesized that these proteins are required for fertility and are thus unique non-hormonal male contraceptive targets that may serve the dual purpose as direct contraceptive targets as well as indirect targets for the degradation of other testis-specific contraceptive targets. In the present study, we have chosen to determine the fertility requirement of these evolutionarily conserved testis-enriched ubiquitin–proteasome pathway proteins (RNF133, RNF148, RNF151, and ZSWIM2) using a CRISPR/Cas9 knockout mouse strategy.

## Results

### Bioinformatic analysis to identify putative E3 ubiquitin-protein ligases

**Fig. 1:**
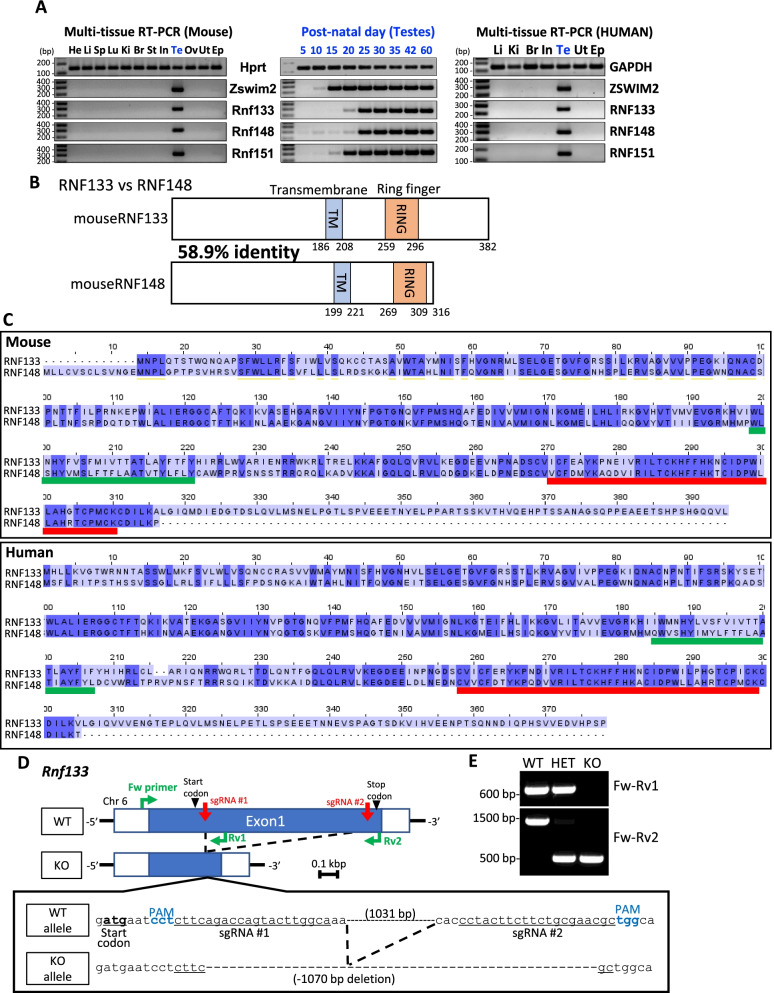
RNF133 is a testis-specific E3 ubiquitin ligase. **A** (Left and right), multi-tissue RT-PCR expression analysis of *Zswim2*, *Rnf133*, *Rnf148*, and *Rnf151* in mice and humans, respectively. *Hprt* and *GAPDH* were used as expression controls. (Middle) RT-PCR from mouse testes at various postnatal days. *Hprt* was used as an expression control. **B** Protein domain structures of mouse RNF133 protein (top) and mouse RNF148 protein (bottom). **C** Sequence alignment of proteins of mouse RNF133 and RNF148 (top), and human RNF133 and RNF148 (bottom). Identified amino acids are highlighted in dark blue. The green and red underbars indicate the transmembrane region and the RING finger domain, respectively. **D** Genomic structure and strategy of generating KO of mouse *Rnf133*, and the genetic sequences deleted by the CRISPR/Cas9 system. **E** Genotyping of *Rnf133* alleles. Primers shown in **D** amplify specific amplicons for the WT (Fw-Rv1) or KO (Fw-Rv2) allele

We used multiple bioinformatics and gene expression approaches to uncover testis-enriched genes that are components of the ubiquitin–proteasome pathway. We searched in-house and published RNAseq data lists [12] and identified four putative RING family E3 ubiquitin ligases (RNF133, RNF148, RNF151, and ZSWIM2). To confirm the expression profiles of *Rnf133*, *Rnf148*, *Rnf151*, and *Zswim2*, we performed RT-PCR of mouse and human reproductive and non-reproductive tissues, confirming that all four E3 ubiquitin ligases are testis-specific in mice and humans (Fig. 1A left and right panels, Additional file 1: Table S1). To glean insight into the potential spermatogenic cell population(s) expressing *Rnf133*, *Rnf148*, *Rnf151*, and *Zswim2*, we performed RT-PCR of mouse testes isolated at postnatal day (P) 5, a timepoint enriched for gonocytes transitioning to Type A spermatogonia, P10 (early, Leptotene, spermatocytes), P15 (late, Pachytene, spermatocytes), P20 (early, round, spermatids), and P30, 35, 42, and 60, which all display fully mature elongated spermatids, and either one complete wave, or multiple waves, of spermatogenesis [13]. As shown in Fig. 1A (center panel), *Rnf133* and *Rnf148* are expressed at day 25, corresponding to the period when round spermatids are transitioning to elongating spermatids, *Rnf151* is expressed at day 20, at the round spermatid stage, and *Zswim2* is expressed abundantly at day 15, towards the end of meiosis. Based on these results, we suspect that all four genes could function during spermiogenesis or in sperm formation and/or for sperm function. Considering the correspondence of the expression pattern of *Rnf133* and *Rnf148* in mouse, we compared the domain structures of those two proteins in mouse and human. In silico prediction reveals that both RNF133 and RNF148 have one transmembrane region and one RING finger domain after the transmembrane region and localized in the cytoplasm (Fig. 1B). Typically, E3 transmembrane proteins are localized to the ER, which our immunostaining for recombinantly expressed human RNF133 confirms (Additional file 2: Fig. S1) [8]. In addition, RNF133 and RNF148 share 58.9% and 54.9% identity in mouse and human, respectively, based on pairwise sequence analysis (Fig. 1C).

### Generation of testis-specific ubiquitin ligase gene knockout mice

We generated knockout mice using the CRISPR/Cas9 system to examine the function of these proteins in vivo. The two gRNAs for each gene were designed as shown in Fig. 1D and Additional file 3, 4, 5, 6: Figs. S2-4, Table S2. One-cell stage embryos were electroporated with Cas9 protein and sgRNAs that were designed to produce large deletions of the coding region of each gene, and the two-cell stage embryos were then transferred into the oviducts of pseudopregnant females. For each gene, the electroporation resulted in deletions of more than 50% of the open reading frame (ORF) in *Rnf133*, *Rnf148*, *Rnf151*, and *Zswim2* (Fig. 1D and Additional file 3, 4, 5: Figs. S2-4), which leads to loss of their functional zinc finger domains. After the deletions in each gene were confirmed by direct sequencing analysis, specific primers for the wild-type (WT) or KO allele were designed and used for genotyping (Fig. 1D, E and Additional file 3, 4, 5: Figs. S2-4). The KO mice containing mutations in each gene did not show any obvious developmental abnormalities or differences in sexual behavior.

Because the *Rnf133* and *Rnf148* genes were closely linked on mouse chromosome 6 by 11.01 centimorgan and structurally similar (Fig. 1B), we could not interbreed single mutants. To generate double KO (DKO) of *Rnf133* and *Rnf148*, *Rnf133* KO female, and *Rnf133* heterozygous (HET) male mice were subjected to in vitro fertilization (IVF), and the zygotes from this IVF were electroporated with gRNAs, to target the *Rnf148* locus. The founder female mice, whose genotype showed *Rnf133* KO and a confirmed deletion in the *Rnf148* gene, were intercrossed to obtain subsequent generations of *Rnf133/Rnf148* DKO mice. To generate *Rnf148*/*Rnf151* DKO and *Rnf148*/*Rnf151/Zswim2* triple KO (TKO) mice, mutant mice of each gene were intercrossed to obtain the required mutant alleles.

### *Rnf133* KO and *Rnf133/Rnf148* DKO males show subfertility, while *Rnf148*, *Rnf151*, and *Zswim2* KO males retain their fertility

**Fig. 2:**
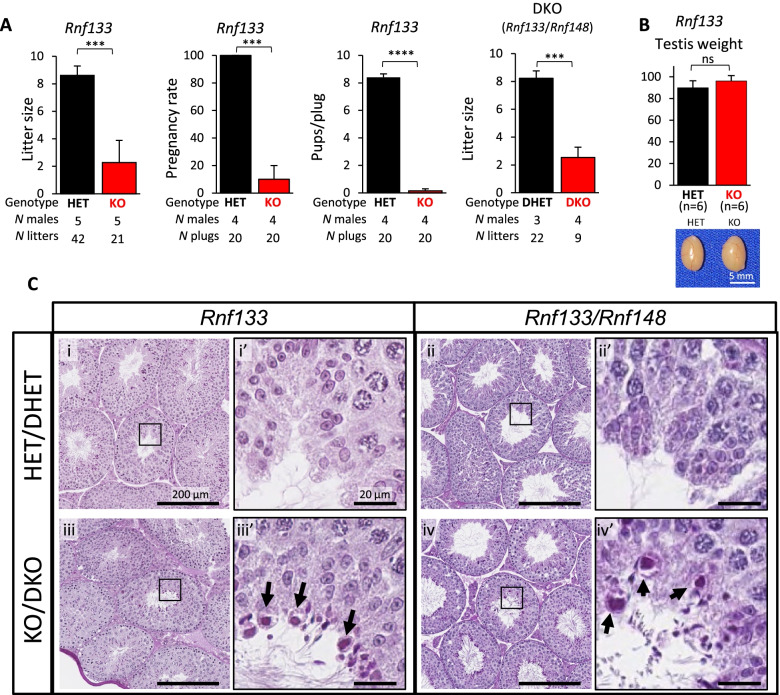
*Rnf133* KO males and *Rnf133*/*Rnf148* DKO males show severe subfertility. **A** Average litter sizes from natural mating (left), pregnancy rate from timed-control mating (second from left), and average pups/copulation plug from timed-control mating (third from left) of *Rnf133* heterozygous (HET) and homozygous knock out (KO). Right: Average litter sizes from natural mating of *Rnf133/Rnf148* double HET (DHET) and *Rnf133*/*Rnf148* double KO (DKO) males. Litter size was measured by the number of pups. *n* ≥ 3 mice/genotype. Data are expressed as the mean ± SEM. Individual data values for each replicate are provided in Additional file 18: Raw data. **B** Average weight and gross images of testis from *Rnf133* HET and KO male mice. *n* = 6 mice/genotype. Data are expressed as the mean ± SEM. Scale bar = 5 mm. **C** (i, ii, iii, and iv) Representative periodic acid-Schiff staining testes from *Rnf133* HET, KO, *Rnf133/Rnf148* DHET, and DKO male mice. Scale bar = 200 μm. (i’, ii’, iii’, and iv’) Representative periodic acid-Schiff staining testes seminiferous tubules at stage IX from testes of *Rnf133* HET, KO, *Rnf133/Rnf148* DHET, and DKO male mice. The magnified images are shown in black squares on (i, ii, iii, and iv) respectively. Scale bar = 20 μm. Black arrows indicate retained sperm nuclei surrounded by excess cytoplasm. This experiment was replicated with three mice per genotype

To assess the functions of RNF133 in vivo, sexually mature *Rnf133* HET or KO males were housed with two WT females for 4 months. The average number of offspring per litter was counted. Five *Rnf133* heterozygous mutant mating pairs had 8.6 ± 0.7 pups per litter on average, whereas five homozygous KO males showed 2.3 ± 1.6 pups per litter on average (Fig. 2A). These data demonstrate that RNF133 has an important role in male fertility. Meanwhile, the *Rnf148* KO, *Rnf151* KO, *Zswim2* KO, *Rnf148/Rnf151* DKO, and *Rnf148/Rnf151/Zswim2* TKO males showed comparable litter size with control male mice (Additional file 7: Fig. S5).

To further evaluate the subfertility of *Rnf133* KO males, timed-matings with WT females were conducted. We counted 15 (HET) or 20 (KO) copulation plugs in total. *Rnf133* HET males produced a successful pregnancy rate (litters/copulation plugs) for 100 ± 0% of copulation plugs, while *Rnf133* KO males had only 10.0 ± 10% pregnancy success (Fig. 2A). The *Rnf133* KO males also fathered significantly fewer pups/plug (0.15 ± 0.15) compared with HET males (8.4 ± 0.24) (Fig. 2A). This result indicates *Rnf133* KO males’ sperm are less likely to successfully fertilize oocytes, resulting in a failed pregnancy and dramatically reduced fecundity per coitus.

Based on the similarity of RNF133 and RNF148 proteins, we hypothesized that RNF148 compensates in the absence of RNF133 in vivo. Mating analysis using *Rnf133*/*Rnf148* DKO male mice revealed that their subfertility remained at a similar level as *Rnf133* single KO males (2.5 ± 0.15 pups, Fig. 2A right). In contrast to *Rnf133* KO and *Rnf133*/*Rnf148* DKO males, *Rnf148*, *Rnf151*, or *Zswim2* single KO, *Rnf148/Rnf151* DKO, and *Rnf148/Rnf151/Zswim2* TKO males showed similar fertility as control males (Additional file 7: Fig. S5).

To characterize the etiology of subfertility of *Rnf133* KO males and *Rnf133*/*Rnf148* DKO male mice, we analyzed testes from *Rnf133* HET, KO, and *Rnf133*/*Rnf148* DKO mice. Histologically, we found that spermatogenesis was grossly normal in *Rnf133* KO and *Rnf133/Rnf148* DKO mice in comparison to heterozygous controls (Fig. 2B and Additional file 8: Fig. S6A). The testes of the *Rnf133* KO and the *Rnf133/Rnf148* DKO were nearly identical to each other and showed a significant increase in sperm nuclei surrounded by excess cytoplasm at stage IX, a phenomenon that we observed considerably less in the control (HET/double HET(DHET)) testes (Fig. 2C). We quantified this occurrence and found that the percentage of sperm nuclei surrounded by excess cytoplasm at stage IX, out of all tubules at stage IX, was 20.5% in the control versus 80.6% in the *Rnf133* KO and 89.2% in the *Rnf133/Rnf148* DKO.

**Fig. 3:**
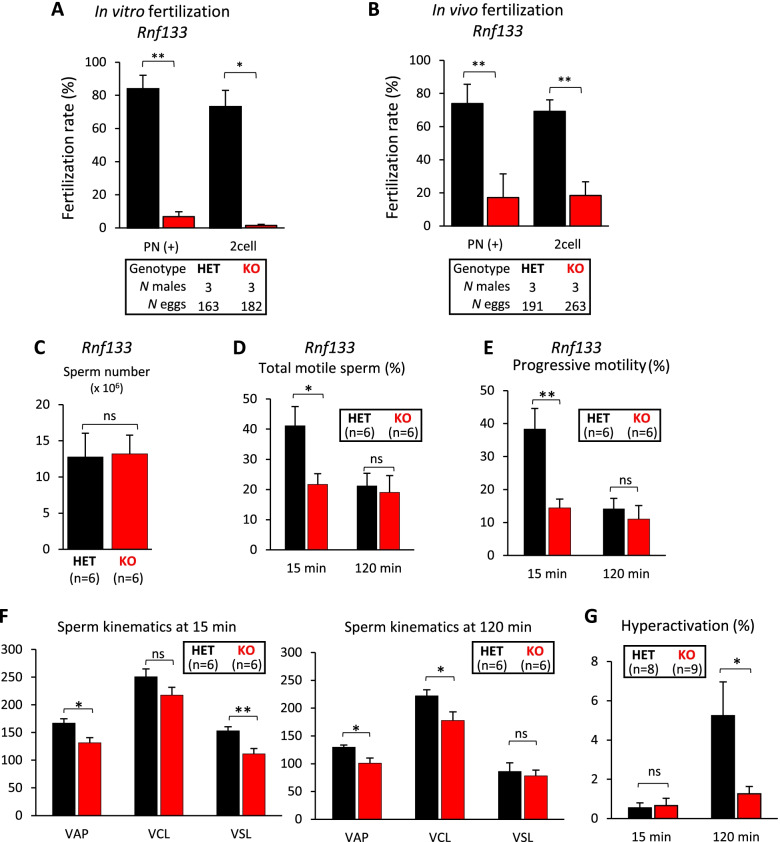
Sperm from *Rnf133* KO males and *Rnf133*/*Rnf148* DKO males demonstrate fertilization and motility defects. **A** Quantification of pronucleus-positive or two-cell embryos from IVF using sperm from *Rnf133* HET and KO males and oocyte from WT females. *n* = 3 mice/genotype, and the data are expressed as the mean ± SEM. **B** Quantification of pronucleus-positive or two-cell embryos from in vivo fertilization by mating *Rnf133* HET and KO males to WT females each. *n* = 3 mice/genotype, and the data are expressed as the mean ± SEM. **A,B** Individual data values for each replicate are provided in Additional file 18: Raw data. **C** Average sperm number from caudal epididymis of *Rnf133* HET and KO male mice. **D** Total motile sperm rate measured by CASA for *Rnf133* HET and KO sperm. **E** Progressive motility rate measured by CASA for *Rnf133* HET and KO sperm. **F** Sperm kinetics after 15 min (left) and 120 min (right) incubation in capacitation medium for *Rnf133* HET and KO sperm. VAP, average path velocity; VCL, curvilinear velocity; VSL, straight-line velocity. **C,D** *n* = 6 mice/genotype, and the data are expressed as the mean ± SEM. **G** Hyperactivated sperm rate classified by CASAnova after 15 min and 120 min incubation in capacitation medium for *Rnf133* HET and KO sperm. *n* ≥ 8 mice/genotype, and the data are expressed as the mean ± SEM

To define the cause of the fertility defects in the *Rnf133* KO mice, we performed IVF with sperm from *Rnf133* HET and KO mice and oocytes from WT female mice (Fig. 3A). In contrast to sperm from *Rnf133* HET controls which fertilized 83.9 ± 8.2% of oocytes and resulted in 73.1 ± 10.0% 2-cell stage embryos (2cell), the sperm from *Rnf133* KO mice only fertilized 6.8 ± 3.0% of oocytes and resulted in 1.4 ± 0.7% 2-cell stage embryos. Similar defects were also observed for in vivo fertilization with WT female mice in which the fertilization rate by *Rnf133* KO sperm was significantly lower than corresponding controls: 17.2 ± 4.1% pronuclei positive (PN +) and 18.4 ± 4.4% 2-cell stage embryos (2cell) from *Rnf133* KO sperm, versus 73.9 ± 11.7% PN + and 69.2 ± 11.3% 2cell from *Rnf133* HET sperm (Fig. 3B). To further understand the origin of this fertilization defect, we examined the number and motility of sperm-derived from the cauda epididymis. The sperm counts from the cauda epididymis between *Rnf133* HET and KO mice showed no significant differences (Fig. 3C). However, when we examined cauda epididymal sperm using computer-assisted sperm analysis (CASA), the percentage of motile sperm and amount of progressive motility were significantly decreased in *Rnf133* KO mice after 15 min incubation (21.7 ± 3.5% and 14.5 ± 2.7%, respectively) compared to HET (41.1 ± 6.4% and 38.4 ± 6.2%, respectively) (Fig. 3D, E). After 120 min incubation, *Rnf133* KO sperm showed no difference in total motile sperm or progressive motility (19.1 ± 5.5% and 11.0 ± 4.1%, respectively) in comparison to *Rnf133* HET sperm (21.2 ± 4.2% and 14.2 ± 3.1%, respectively). The velocity parameters (average path velocity: VAP, curvilinear velocity: VCL, straight-line velocity: VSL) were impaired in *Rnf133* KO sperm. The decrease in VAP (HET, 167.1 ± 7.7 vs. KO, 131.7 ± 9.3) and VSL (HET, 153.1 ± 7.3 vs. KO, 111.4 ± 9.9) at 15 min of incubation, and VAP (HET, 129.6 ± 3.9 vs. KO, 101.0 ± 9.2) and VCL (HET, 222.0 ± 11.2 vs. KO, 177.9 ± 15.3) at 120 min of incubation were significantly different (Fig. 3F). Likewise, although *Rnf133*/*Rnf148* DKO mice showed no significant difference from *Rnf133/Rnf148* double HET (DHET) in testicular size, weight, and caudal epididymal sperm, we found significant decreases in the percentage of motile sperm (15 min, DHET, 64.6 ± 4.5% vs. DKO, 36.9 ± 2.1%; 120 min, DHET, 43.9 ± 2.6%, DKO, 24.8 ± 5.3%) and progressive motility (15 min, DHET, 60.1 ± 4.6% vs. DKO, 30.9 ± 2.4%; 120 min, DHET, 25.0 ± 3.3%, DKO, 13.0 ± 3.9%) (Additional file 8: Fig. S6A-E).

To evaluate the rate of sperm hyperactivation, which is a critical factor for fertilization success, we utilized the machine learning algorithm CASAnova [14]. The percent of hyperactivated motile *Rnf133* HET sperm incubated in capacitation medium increased ~ tenfold, from 0.5 ± 0.3% at 15 min to 5.2 ± 1.7% after 120 min, while *Rnf133* KO sperm did not show a dramatic change in hyperactivation (0.7 ± 0.4% at 15 min and 1.3 ± 0.4% at 120 min) (Fig. 3G). Likewise, the hyperactivation rate between DHET and *Rnf133*/*Rnf148* DKO sperm was not statistically different at 15 min (DHET, 0.3 ± 0.1% vs. DKO, 0.3 ± 0.2%) but a difference was observed at 120 min (DHET, 3.8 ± 0.8%, DKO, 1.6 ± 0.3%) (Fig. S6F). There was no significant difference between the sperm hyperactivation rates of the *Rnf133* KO and the *Rnf133/Rnf148* DKO mice (*P* = 0.69 at 15 min, *P* = 0.24 at 120 min). Our discovery of compromised motility of sperm and the impaired hyperactivation of sperm from *Rnf133* KO and *Rnf133*/*Rnf148* DKO males explains the in vitro and in vivo defects in fertilization and the subfertility in the *Rnf133* KO and *Rnf133/Rnf148* DKO males. Consistent with no significant difference in fertility (Additional file 7: Fig. S5), sperm from the *Rnf148* KO, *Rnf151* KO, and *Zswim2* KO did not show any significant differences in terms of testis weight and sperm parameters in comparison to controls, except VSL at 120 min for *Rnf148* KOs, and VAP and VSL at 120 min for *Rnf148/Rnf151* DKOs. However, as mentioned, this did not affect their fertility in natural mating with WT female mice (Additional file 7, 9, 10, 11, 12: Fig. S5, S7-S10).

### *Rnf133* KO results in aberrant head-neck morphology

**Fig. 4:**
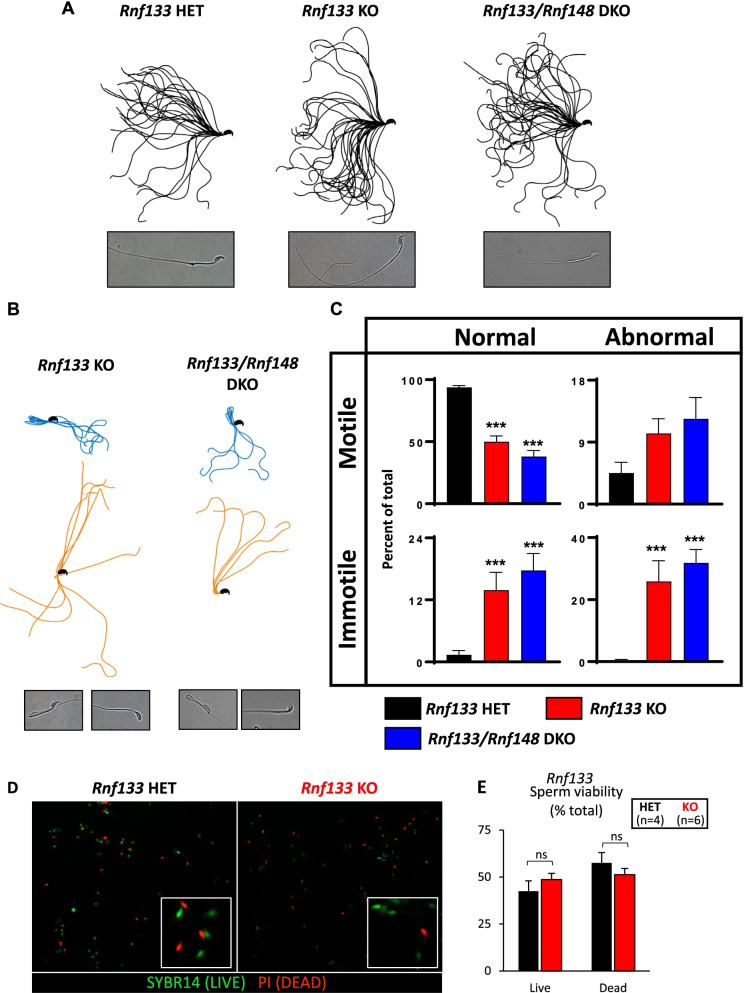
*Rnf133* KO and *Rnf133/148* DKO sperm show abnormal morphology that correlates with immotility. **A** Flagellar waveform patterns of *Rnf133* HET and KO, and *Rnf133/148* DKO sperm. *n* = 3 mice/genotype. Black lines indicate traces from motile sperm. The representative images of *Rnf133* HET and KO and *Rnf133/148* DKO sperm are shown at the bottom. **B** Flagellar waveform patterns of abnormal sperm from *Rnf133* KO and *Rnf133/148* DKO. *n* = 3 mice/genotype. Blue and orange lines indicate sperm bent at the principal piece and at the neck, respectively. **C** Average percentages of motile and immotile sperm with normal or abnormal morphology are presented for the *Rnf133* HET, *Rnf133* KO, and *Rnf133/148* DKO mice. *n* = 3 mice/genotype. **D** Representative multichannel fluorescence staining of *Rnf133* HET and KO sperm incubated with membrane-permeant SYBR14 (green) and membrane-impermeant propidium iodide (PI; red) nucleic acid stains to identify live (SYBR14 + /PI −) and dead (SYBR-/PI +) sperm. *n* = 4 mice/genotype. **E** Results from flow cytometry-based quantification of the percentage of live (SYBR14 + /PI − and SYBR + /PI +) and dead (SYBR − /PI +) *Rnf133* HET and KO sperm. SYBR + /PI + cells mark a population of cells that were alive when removed from the animal but are rapidly undergoing death in vitro. *n* = 4 mice/genotype, and the data are expressed as the mean ± SEM

To characterize the cause of the impaired motility in *Rnf133* KO males, we analyzed the waveform of the flagella of sperm (Fig. 4A). Tracing of sperm flagella after 15-min incubation revealed that *Rnf133* KO and *Rnf133/148* DKO males had sperm cells with aberrant morphologies such as bending at the head/midpiece and bending at the midpiece/principal piece (Fig. 4B), which are rarely observed in *Rnf133* littermate controls. Since we observed a significant decrease in total motile and progressively motile sperm in *Rnf133* KO and *Rnf133/148* DKO in comparison to controls (Fig. 3D, E and Additional file 8: Fig. S6C, D), we next examined the relationship between aberrant morphology and change in motility. Indeed, when morphology and motility were co-quantified in *Rnf133* KO and *Rnf133*/*Rnf148* DKO sperm in comparison to controls, we found statistically significant relationships for most subsets of sperm. As shown in Fig. 4C, there was a statistically significant 1.9- and 2.5-fold decrease in the percentage of normal morphology motile sperm in *Rnf133* KO (50 ± 4.6%) and *Rnf133/148* DKO (38 ± 4.8%) in comparison to controls (93.7 ± 1.6%). Additionally, a statistically significant 10.1-fold and 12.8-fold increase in the percentage of normal morphology immotile sperm was observed in KO (13.9 ± 3.4%) and DKOs (17.7 ± 3.3%) in comparison to controls (1.4 ± 0.8%), and a statistically significant 67-fold and 82-fold increase in the percentage of abnormal morphology immotile sperm in KOs (25.9 ± 6.6%) and DKOs (31.9 ± 4.3%) in comparison to controls (0.4 ± 0.3%) (all *P* < 0.001, as calculated through one-way ANOVA). To confirm that the increased immotility does not result from an increase in sperm cell death, we performed a live/dead cell assay and found no significant difference between *Rnf133* KO and *Rnf133*/*Rnf148* DKO sperm in comparison to controls (Fig. 4D,E and Additional file 8: Fig. S6G-I). These data suggest that the aberrant shape of *Rnf133* KO and *Rnf133/148* DKO sperm is the major cause of the reduced motility and hyperactivation in these mutant sperm, although other additional defects such as abnormal sperm head morphology may also be possible.

### RNF133 is an E3 ligase that interacts with the ER-localized E2 enzyme UBE2J1

**Fig. 5:**
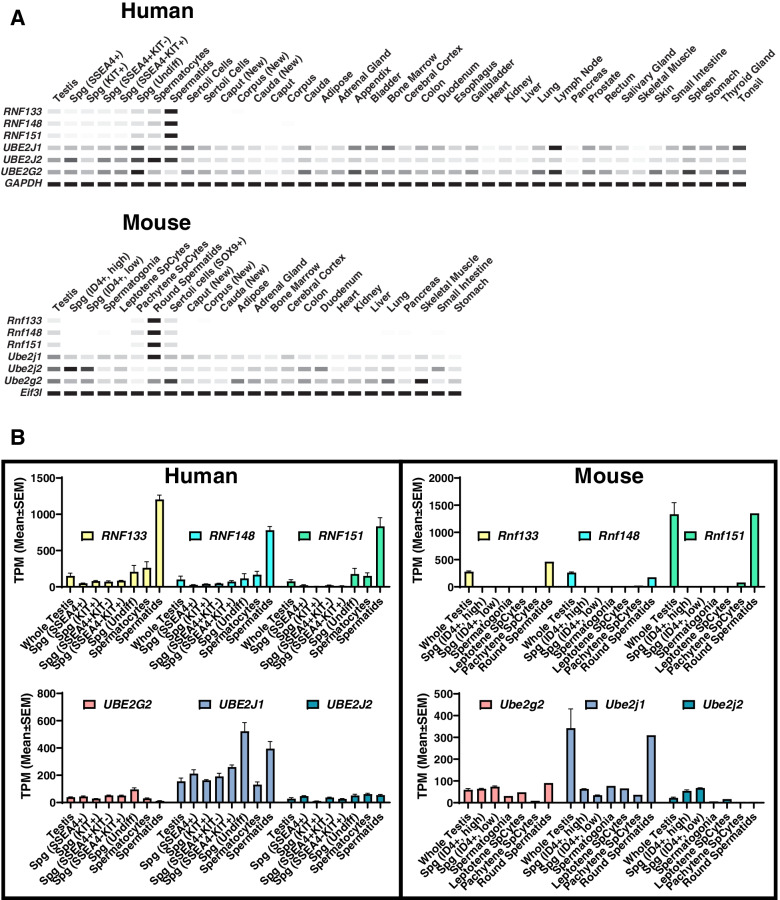
In silico analysis of E2 enzymes. **A** The digital PCR (heatmap) depicts the average transcripts per million (TPM) value per tissue per gene from the indicated human and mouse RNAseq datasets. White = 0 TPM, Black = maximum TPM value for that gene across all tissues in that species. *GAPDH* and *Eif3l* are included as references. **B** Bar graphs of the testis and germ cell dataset average TPM values depicted in **A**. The number of replicates per sample are as described in Robertson et al. [12]

**Fig. 6:**
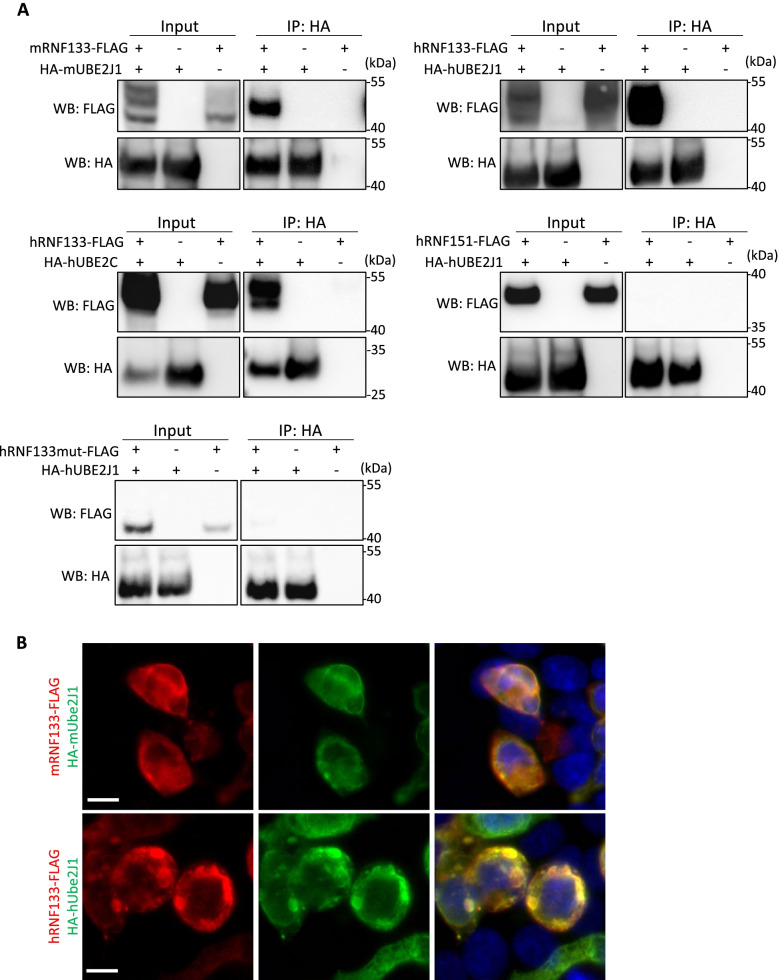
RNF133 interacts with UBE2J1. **A** Western blot analysis of immunoprecipitation with exogenous mouse/human RNF133-FLAG and HA-UBE2J1, human UBE2C, human RNF151, and human RNF133-mutant-FLAG in HEK293 cells. The anti-HA antibody was used for immunoprecipitation and the anti-FLAG antibody was used for Western blot analysis. This experiment was replicated three times and representative blots are presented. **B** Immunofluorescent staining with exogenous mouse/human RNF133-FLAG and HA-UBE2J1 in HEK293 cells. The anti-FLAG antibody and the anti-HA antibody were used for RNF133 (red) and UBE2J1 (green), respectively. Scale bar, 10 µm. This experiment was replicated three times and representative images are presented

E3 ubiquitin ligases function with E2 ubiquitin-conjugating enzymes in the ubiquitylation process. To identify the E2 protein that interacts with RNF133, we first conducted in silico analysis using our recently published RNAseq data (Fig. 5A,B) [12]. Based on our findings, we decided to focus on *Ube2g2*, *Ube2j1*, and *Ube2j2*, which are all conserved in mouse. As seen in Fig. 5A, B, *Ube2g2 Ube2j1*, and *Ube2j2* are all expressed in testis and testicular germ cells, with *Ube2j1* showing the highest levels of expression in testis and testicular germ cells in both humans and mice (Fig. 5A,B). Moreover, UBE2J1 is an ER-localized transmembrane protein and has been reported to have a critical role in ER quality control during spermiogenesis, with *Ube2j1* KO mice displaying a similar phenotype to our *Rnf133* KO and *Rnf133/Rnf148* DKO [9]. Thus, we hypothesized that RNF133 interacts with UBE2J1. Using HEK293 cells, FLAG-tagged mouse or human RNF133 and HA-tagged mouse or human UBE2J1 were co-expressed and subjected to immunoprecipitation analysis to detect their complexes (Fig. 6A and Additional file 13, 14: Fig. S11, S12). Total proteins were extracted from transfected cells, and each protein sample was pulled down using anti-HA beads. Input lysate and pull-down samples were subjected to SDS-PAGE, and immunoblots were incubated with anti-FLAG antibody. We confirmed robust expression of FLAG and HA in each sample transfected with the vectors encoding RNF133-FLAG and/or HA-UBE2J1 (Fig. 6A and Additional file 13, 14: Fig. S11, S12) and found both mouse and human FLAG-tagged RNF133 interact with HA-tagged UBE2J1 (Fig. 6A, and Additional file 13, 14: Fig. S11, S12). Interestingly, although we observed interaction between HA-UBE2C and RNF133-FLAG (Fig. 6A and Additional file 13, 14: Fig. S11, S12), interaction between HA-UBE2J/RNF151-FLAG was not detected (Fig. 6A and Additional file 13, 14: Fig. S11, S12). To verify that the robust interaction between RNF133 and UBE2J1 is not an artifact, we created a human RNF133 construct in which the RING finger domain was deleted (RNF133-mutant) and replicated the co-immunoprecipitation (IP) assay using this human RNF133-mutant and UBE2J1. Although there was a faint interaction between RNF133-mutant and UBE2J1, possibly due to both proteins localizing at the ER membrane and/or because other regions of RNF133 and UBE2J1 interact normally, the intensity of the signal in the immunoprecipitated sample with UBE2J1 was reduced in RNF133-mutant compared to RNF133 wild-type. As a numerical analysis, we calculated a ratio based on the band intensity of the IP lane to the input lane for each RNF133:UBE2J1 interaction and confirmed that human RNF133-WT:UBE2J1 or mouse RNF133-WT:UBE2J1 had an ~ sevenfold or ~ 16-fold IP:input ratio, respectively, compared to human RNF133-mutant:UBE2J1 IP:input ratio (human RNF133-WT, 1.74 vs. mouse RNF133-WT, 3.95 vs. RNF133-mutant, 0.24) (Fig. 6A and Additional file 15: Fig. S13).

In parallel, transfected HEK293 cells were stained with the anti-FLAG antibody for RNF133, and with the anti-HA antibody for UBE2J1. We confirmed the co-localization of both human and mouse RNF133 and UBE2J1 in the ER in vitro (Fig. 6B). These data strongly support the interaction of RNF133 and UBE2J1 during spermiogenesis in mice and humans.

### Electron microscopic analysis of *Rnf133* KO sperm resembles the *Ube2J1* KO phenotype

**Fig. 7:**
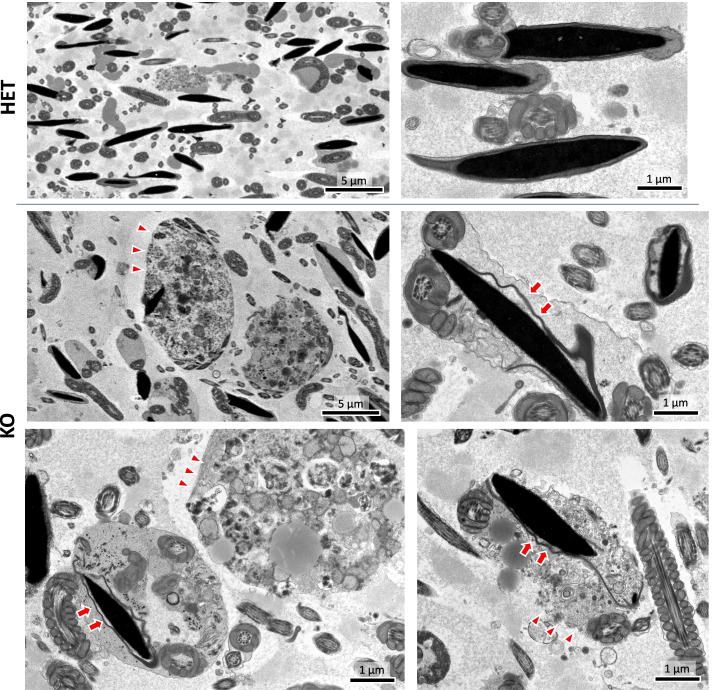
EM images of *Rnf133* HET/KO sperm in the epididymis. *Rnf133* KO sperm have cytoplasm around normal-shaped nuclei (arrows) and retain abnormally massive cytoplasm involving vacuoles and organelles (arrowheads). This experiment was replicated with a minimum of three mice per genotype, and representative images are shown

Since we discovered abnormal sperm morphology with bent neck/flagella in *Rnf133* KO mice and confirmed RNF133 interaction with UBE2J1 in vitro, we performed transmission electron microscope (TEM) analysis of epididymal tissue to assess the morphology of KO sperm at the ultrastructural level. Observation of caudal epididymis from *Rnf133* KO mice revealed that although *Rnf133* KO sperm had morphologically normal nuclei, the cytoplasm around the nuclei and neck was retained and contained organelles such as the endoplasmic reticulum and vacuoles (Fig. 7) Similar abnormalities were shown previously in *Ube2j1* KO sperm [9]. These observations robustly suggest that RNF133 and UBE2J1 function together in a ubiquitination pathway to degrade and discard unnecessary proteins from the ER.

## Discussion

In this study, we first discovered that RNF133 is critical for male fertility in mice by generating a novel *Rnf133* KO mouse model. RNF133 is a testis-specific ubiquitin ligase E3 enzyme whose absence causes severe subfertility due to abnormal sperm morphology resulting in impaired sperm motility. Through immunoprecipitation and immunostaining, we found that RNF133 interacts with UBE2J1, which is an E2 ubiquitin-conjugating enzyme that localizes to the ER membrane. UBE2J1-deficient sperm have previously been shown to have defects in removing cytoplasm from elongating spermatids, in addition to flagella and acrosome function defects [9]. Our results demonstrate that our *Rnf133* KO male mice phenocopy many of the reproductive defects in *Ube2J1* KO mice. Considering that UBE2J1 has been postulated to function in ERAD [15], our results also suggest that RNF133 likely serves a critical and complementary role in ER quality control during spermiogenesis. These findings allow us to hypothesize that UBE2J1 and RNF133 are the first ER-localized E2 and E3 transmembrane proteins, respectively, that function in ERAD during spermiogenesis and for the formation of normal spermatozoa.

During spermiogenesis, spermatozoa are dramatically remodeled architecturally into a morphology required for proper fertilization [16]. This includes mitochondrial rearrangement around the flagella at the midpiece of the tail, and proteins, organelles, and bulk cytoplasm that are no longer needed are discarded through the extrusion of cytoplasmic droplets, which are eventually removed from the sperm head and neck region [17, 18]. Our previous studies have shown that spatiotemporal organelle migration is critical for successful spermatogenesis [19]. Similarly, improper retainment of cytoplasmic droplets during spermiogenesis has severe consequences on sperm maturation in the epididymis. Although the correlation between ERAD and spermatogenesis is almost impossible to demonstrate in vivo, Koenig et al. provided evidence that UBE2J1, a well-characterized component in the ERAD system in vitro, is critical for spermiogenesis [9]. The authors hypothesized that spermatogenic failure is attributed to the accumulation of misfolded proteins in the ER causing excess residual cytoplasm in elongating spermatids. Moreover, UBQLN1, which shuttles proteins to the proteasome during ERAD [20, 21], was reported to interact with SPEM1, which is required for processing of cytoplasmic droplets during spermatogenesis and male fertility [22, 23]. Our *Rnf133* KO mouse model further suggests the importance of ERAD during spermatogenesis and the correlation between ERAD failure and unsuccessful spermatogenesis.

It remains unclear why *Rnf133* KO are subfertile and *Ube2j1* KO mice are infertile, and it is unknown which target proteins interact with UBE2J1 and RNF133 for degradation through ERAD. RNF133 may not be the only ER-localized E3 enzyme that interacts with UBE2J1. Despite ~ 50% sequence identity between RNF133 and RNF148 proteins (Fig. 1C), and a potential similarity in function, RNF148 did not compensate for the absence of RNF133 in mice (Figs. 2, 3, and 4 and Additional file 8, 9: Fig. S6, S7). In light of these results, we suspect that RNF148 does not function with UBE2J1, at least in mice, and is not required for spermiogenesis (Additional file 9: Fig. S7). The report of loss of function of GRTH/DDX25 mouse model for human azoospermia patients showed infertility due to failure of round spermatid elongation [24]. In this model, the reduction in transcripts of the ubiquitination-related gene, including *Ube2j1*, *Rnf8*, *Rnf133*, and *Rnf138*, was observed. Although these other RNFs are not transmembrane-type proteins, they may interact with UBE2J1 and have compensatory regulations for the absence of RNF133. Alternatively, the function of RNF8, which is to mediate ubiquitination of H2A on the sex chromosomes in meiosis, could suggest that UBE2J1 acts as a histone modulator in addition to functioning in a putative ERAD pathway and thereby explain the phenotypic conflict with the *Rnf133* KO mouse model [25]. Also, the high mortality and low growth rate at juvenile stages of *Ube2j1* KO males might have a delayed-onset effect on their fertility [9]. Future proteomic studies on the target proteins of RNF133 and/or UBE2J1 will unmask more of the putative ERAD signaling network during spermatogenesis.

The need for a non-hormonal male contraceptive has been widely recognized, as the global human population continues to grow at an unsustainable rate reaching 7.8 billion people in 2020 (UN, World Population Prospects). This continued population increase is resulting in changes in our climates and ecosystems and is predicted to eventually lead to global shortages of food, water, and other resources for future generations [26]. Since the development of the birth control pill for women, there have been few advances in contraceptive development [27], including small molecule-based contraceptives available to men [28]. To curb rampant population growth and its dire consequences, we require new, safe contraceptive options that specifically target the male germline. Non-hormonal contraceptives that target testis-specific proteins should reduce or eliminate unwanted side effects. PROTACs are a promising inhibitory approach for fertility-involved gene products. PROTACs are a relatively new technology wherein a small molecule binding the target protein is conjugated to a so-called degrader, a small molecule capable of recruiting an E3 ligase complex such that polyubiquitination and, ultimately, proteolysis of the target occurs due to induced proximity of the E3 ligase complex [29–31].

Although the *Rnf133* KO mouse model showed significant fertility defects due to abnormal sperm head shape and irregular motility (Figs. 2, 3, and 4), RNF133 protein is likely not a suitable direct male contraceptive target as its ablation in males results in subfertility rather than complete infertility, the latter of which is the desired outcome of an effective non-hormonal male contraceptive. However, despite this, as RNF133 is involved in the ubiquitin-proteosome pathway, RNF133 has the potential to be a component target of PROTACs that function as male contraceptives. Our studies herein have therefore provided novel insights into male infertility resulting in combined asthenozoospermia and teratozoospermia, and the identification of future diagnostic markers of infertility in men.

## Conclusions

In summary, our studies have defined the fertility essential roles of four testis-specific or enriched ubiquitination-proteasome pathway proteins RNF133, RNF148, RNF151, and ZSWIM2, and clarified the fundamental ubiquitin–proteasome pathway components specific to germ cells. Our study has fully assessed the reproductive requirement of RNF133 demonstrating that it is a crucial transmembrane protein in the ubiquitin-proteosome pathway in germ cells. RNF133 has a unique function operating within the cytosol of testicular germ cells [10], ensuring unaffected (average) coitus frequency in *Rnf133* KO males. In the future, RNF133 may be uniquely exploited through PROTACs to generate an innovative and potentially effective non-hormonal germ cell-directed male contraceptive.

## Methods

### RT-PCR

mRNA was collected from tissues of C57BL6J/129SvEv hybrid mice and reverse transcribed into cDNA. Human multiple tissue cDNA was purchased from BD Biosciences. RT-PCR was performed with mouse or human cDNA using the primers in Additional file 1: Table S1 as described previously [32].

### Protein bioinformatic analysis

Bioinformatic analysis of RNF133 and RNF148 was performed as previously reported [33]. Protein sequences of mouse and human were collected and aligned using Clustal Omega multiple sequence alignment (www.ebi.ac.uk/Tools/msa/clustalo) [20]. Alignment summary graphics were created using the Jalview program (www.jalview.org/getdown/release) [21]. The SMART protein domain annotation resource (http://smart.embl-heidelberg.de) [22] was used to analyze protein domain architecture. Pairwise protein sequence analysis was conducted using the SIM alignment tool (https://web.expasy.org/sim).

### Animals

B6D2F1 mice were purchased from Charles River Labs (Wilmington, MA, USA) and used for generating *Rnf133*, *Rnf148*, *Rnf151*, and *Zswim2* mutant founder mice. In-house hybrid mice (C57BL/6 J × 129S5/SvEvBrd) were mated with mice heterozygous for each mutant gene to expand each line. For phenotypic analysis, sexually mature male mice (6 weeks to 6 months old) were used. All mice were housed with a 12-h light/dark cycle. A minimum of three mice per genotype was used for each experiment.

### Generation of knockout mice

Single-guide RNA (sgRNA) target sequences for each gene were designed using the CRISPRdirect suite (https://crispr.dbcls.jp/) as shown in Additional file 6: Table S2. The custom gRNAs were ordered (Sigma) and assembled into a ribonucleoprotein (RNP) complex with Cas9 protein (Thermo Fisher Scientific) as described previously [33]. The RNPs were electroporated into zygotes harvested from superovulated B6D2F1 females using an ECM 830electroporation system (BTX, Holliston MA). Embryos were cultured overnight to the 2-cell stage before being transferred into the oviducts of pseudopregnant CD-1 mice (Center for Comparative Medicine, Baylor College of Medicine). Founder mutations in pups born were identified by PCR and Sanger sequencing. Mice were genotyped with the primers shown in Additional file 16: Table S3.

### Male fertility assessment

Male fertility was analyzed as previously described. Sexually mature male mice were continuously caged with two WT of C57BL6J/129SvEv hybrid females for 4 months. During the mating period, the number of pups born per litter and the number of litters per male each month were counted. Average litter sizes are presented as the average number of pups per litter from all the males. Average litters/male is presented as the average number of litters sired per male from all males of each genotype. For timed mating, sexually mature males were continuously caged with two WT females and each mating was confirmed by the observation of a copulation plug. Pregnancy rates are calculated as the average of the number of litters produced divided by the number of plugs counted per male. Litter size is calculated as the average number of pups produced per plug per male of each genotype.

### Histology

Testes were collected and fixed in Bouin’s fixative (Sigma Aldrich) overnight at RT. After washing in 70% ethanol, testes were embedded in paraffin. Five-micrometer sectioned tissues were stained by periodic acid-Schiff (PAS)-hematoxylin stain, followed by histological analysis.

### In vivo and in vitro fertilization analysis

The in vivo fertilization and the IVF experiments were performed as described previously [34]. For in vivo fertilization analysis, sexually matured B6129SF1/J hybrid female mice were superovulated by injection with pregnant mare serum gonadotropin (PMSG) and a follow-up injection of human chorionic gonadotropin (hCG) after 48 h. After injection of hCG, two superovulated females were mated to a single *Rnf133* HET or KO mouse overnight. The following day, zygotes or unfertilized oocytes were collected from females with copulation plugs. Collected zygotes/oocytes were inspected for the presence of two pronuclei and the polar body. Zygotes/oocytes were cultured in KSOM medium overnight in a 37 ℃ incubator under 5% CO_2_ and observed for progression to the two-cell stage. For IVF, oocytes collected from superovulated B6129SF1/J hybrid females were placed in TYH medium supplemented with 4% BSA (Millipore). Epididymal sperm were collected from *Rnf133* HET or KO male mice and incubated in TYH medium for 2 h. Capacitated sperm were inseminated into the drops containing the cumulus-oocyte complex at a final concentration of 1 × 10^5^ sperm/mL. After 5 h of coincubation, the formation of pronuclei was observed. Zygotes/oocytes were cultured in KSOM medium overnight and observed for progression to the two-cell stage.

### Sperm motility analysis

Sperm number and motility were measured as described previously [19]. Sperm were extracted by mincing caudal epididymis and incubated in TYH medium (Millipore) supplemented with 4% BSA under 5% CO_2_ at 37 °C. After 15- and 120-min incubations, sperm samples were applied onto a chamber of 100-µm depth analyzed counting slides (CellVision, Heerhugowaard, Netherlands) and analyzed using the Hamilton Thorne CEROS II system.

### Sperm viability analysis

KO and HET sperm viability was assessed as previously described using the LIVE/DEAD™ Sperm Viability Kit by Thermo Fisher [34]. Caudal epididymal sperm was released into 1.0 mL HEPES-buffered HTF media (mHTF; 101.6 mM NaCl, 4.69 mM KCl, 0.2 mM MgSO_4_, 0.37 mM KH_2_PO_4_, 2.04 mM CaCl_2_, 4.0 mM NaHCO_3_, 21.0 mM HEPES pH 7.4, 2.78 mM glucose, 0.33 mM sodium pyruvate, 21.4 mM sodium lactate, 75 μg/mL penicillin G Na salt, 50 μg/mL streptomycin sulfate, 2 μg/mL phenol red, and 5 mg/mL BSA) at 37 °C. SYBR-14 and propidium iodide dyes were added at the manufacturer’s recommended dilutions and confirmation of fluorescent staining sperm was carried out through multichannel fluorescence microscopy. Quantification of the percentage of live (SYBR14 + /PI- and SYBR + /PI +) sperm and dead (SYBR-/PI +) sperm was carried out through flow cytometry on a BD FACSCanto II using the appropriate fluorophore-negative controls to allow for proper gating of live/dead sperm populations (Additional file 5: Fig. S6G).

### Sperm waveform analysis

The sperm from epididymides were released into TYH medium and placed onto a chamber with a depth of 100 µm. The slides were placed on a heating plate set at 37 ℃ to maintain optimal temperature during video recording. The sperm motility was videotaped at 40.4 frames per second with a ZEISS Axio Observer inverted microscope at × 20 magnification equipped with a high-speed camera (Axiocam 702, Carl Zeiss, Oberkochen, Germany). The obtained data were exported to consecutive images of each frame using a ZEN 2 Blue Edition software (Carl Zeiss) for subsequent sperm waveform analysis and co-quantification of motility and morphology. Frames that captured one or more in-focus sperm displaying the side-profile of the sperm head were used for subsequent repositioning and overlaying of the images, and tracing of the sperm tails. Unless otherwise noted through color-coded tails, composite waveform images (black tails) are derived from motile sperm as determined by observing swimming motion in multiple frames of the video. Color-coded (purple and blue) tails correspond to immotile sperm captured in the video. For motile and immotile sperm, only those sperm displaying the side-profile of the sperm head were used for tracing images. Co-quantification of motility and morphology was performed by counting all in-focus, in-frame sperm of each of the following classifications: motile, normal morphology; motile, abnormal morphology; immotile, normal morphology; and immotile, abnormal morphology. Across all three genotypes, the data are comprised of manual classification of a total of 1242 cells from 2 h and 48 min of videos, across 35 high-power fields (~ 5 min of video per field): HET (10 fields totaling 46 min of video), *Rnf133* KO (10 fields totaling 57 min of video), and *Rnf133/148* DKO (15 fields totaling 65 min of video).

### RNAseq analysis and digital PCR heatmaps

Quantitative heatmaps depicting the average transcripts per million (TPM) value per tissue per gene in a graphical output resembling semi-quantitative PCR was performed as previously described [12]. Briefly, we recently performed a thorough re-analysis of 243 previously published mouse and human reproductive and non-reproductive tissue and cell RNAseq datasets, in parallel with data from 21 new tissues that we processed (NCBI GEO Accession GSE150854). The final dataset is comprised of 3 new (GSE150854) and 5 previously published human testis datasets [35], 27 previously published purified human germ cell datasets [36, 37], 6 previously published purified human Sertoli cell datasets [36, 38], 9 new (GSE150854) and 6 previously published human epididymis segment datasets [39], 6 previously published mouse testis datasets [40], 9 new mouse epididymis datasets (GSE150854), 10 previously published purified mouse germ cell datasets [41, 42], and 3 previously published purified mouse Sertoli cell datasets [43]. An additional 118 previously published datasets contributed to the 26 non-reproductive human tissues [44] and 62 previously published datasets contributed to the 14 non-reproductive mouse tissues [40]. The human and mouse average and standard deviation TPM expression values depicted graphically in Fig. 5A,B can be found in Additional files 3 and 4 (Tables S3 and S4) in Robertson et al. [12].

### Cloning and expression of mouse and human RNF133/UBE2J1/RNF151/UBE2C/RNF133-mutant in HEK293 cells

The full-length open reading frame (ORF) of human/mouse RNF133-FLAG, human/mouse HA-UBE2J1, human RNF151-FLAG, human UBE2C, and human RNF133-mutant-FLAG were cloned from mouse/human testis cDNA and the Kenneth Scott cDNA Clone Collection in Baylor College of Medicine using primers in Additional file 17: Table S4. The primers for cloning human *RNF133*/mouse *Rnf133*/human *RNF151* contained EcoRI restriction sites and Kozak sequence in forward (Fw) primers and C-terminal FLAG tag and NotI restriction sites in reverse (Rv) primers. The primers for cloning human *UBE2J1*/mouse *Ube2J1* contained PstI/EcoRI restriction sites respectively, Kozak sequence, and N-terminal HA tag in Fw primers, and NotI restriction sites in Rv primers. The primers for cloning human *UBE2C* contained EcoRI restriction sites respectively, Kozak sequence, and N-terminal HA tag in Fw primers, and NotI restriction sites in Rv primers. Human RNF133-mutant was designed to delete the entire RING domain region. The primers for cloning the 5′ side of human RNF133-mutant contained EcoRI/EcoRV and the primers for cloning the 3′ side contained EcoRV/NotI. Those constructs were cloned and inserted into the pCAG1.1 mammalian expression vector. The resulting plasmids were transfected into HEK cells using Lipofectamine 3000 (Invitrogen) as the manual instructs. The transfected cells were used for immunoprecipitation and immunofluorescent analysis.

### Co-immunoprecipitation assay

HEK cells were seeded in a 10-cm plate and transfected with each pair of pCAG-human*RNF133*/pCAG-human*UBE2J1*, pCAG-mouse*Rnf133*/pCAG-mouse*Ube2J1* pCAG-human*RNF151*/pCAG-human*UBE2J1*, and human*RNF133*/pCAG-human*UBE2C* using Lipofectamine 3000. The collected cells were lysed using sonication in Pierce IP buffer (25 mM Tris–HCl pH 7.4, 150 mM NaCl, 1 mM EDTA, 1% NP-40, and 5% glycerol) containing a protease inhibitor. Sonication was carried out on a Misonix Qsonica S-4000 Ultrasonic Sonicator Cell Disruptor for 10 s with 1 s ON and 1 s OFF at 20% amplitude. Lysates were cleared by centrifugation at 18,000 × *g* for 3 min at 4 ℃, and supernatants were used for the following immunoprecipitation. The supernatants containing the same amount of protein were incubated with Pierce™ Anti-HA Magnetic Beads (Thermo Scientific™) at 4 ℃ for 1 h. The beads were washed with IP buffer containing 2 mM DTT three times. Washed lysates were incubated in 0.1 M glycine, pH 2.0, and then bound proteins were eluted with LDS sample buffer at 95 °C for 10 min. The samples were then evaluated by immunoblotting with antibodies against FLAG (F7425, Millipore Sigma, USA) or HA (RRID:AB 1,549,585, #3724, Cell Signaling Technology, USA). The chemiluminescent signal was developed using ChemiDoc Imaging System (BioRad, USA) using the same exposure time for each sample set. The quantification of the lanes in the immunoblot analysis was calculated using ImageJ (https://imagej.nih.gov/ij/).

### Immunofluorescence imaging

Glass coverslips (22 × 22 mm) were placed into each well of a 6-well plate and seeded with HEK293 cells. One microgram of each plasmid DNA was mixed and transfected into the cells using Lipofectamine 3000. The plates were incubated at 37 ℃ under 5% CO_2_. After 72 h incubation, the cells were fixed with 4% PFA. After permeabilization with 0.3% Triton-X for 10 min, coverslips were blocked for 1 h at RT in 1% skim milk + 1% normal donkey serum. Coverslips were briefly washed and then incubated with the primary antibodies anti-FLAG (for RNF133-FLAG, F7425, Millipore Sigma, USA), anti-HA (for HA-UBE2J1, #2367, Cell Signaling, USA), and anti-calnexin (10,427–2-AP, Proteintech, USA) diluted in blocking solution overnight at 4 ℃ in a humidified chamber. After washing three times, coverslips were then incubated with fluorophore conjugated secondary antibodies diluted in blocking buffer for 1 h at RT, followed by incubation with DAPI. Coverslips were mounted on slides using Immumount (Thermo Fisher Scientific, USA), and slides were imaged with a Zeiss LSM 880 microscope.

### Transmission electron microscopy (TEM)

Testis samples were fixed in 3% glutaraldehyde. Tissue samples were washed in 1 M sodium phosphate buffer (pH 7.3), post fixed in 1% osmium tetroxide for 1 h, and dehydrated through graded alcohol washes. Tissue samples were infiltrated (hardened) with acetone and Polybed 812 plastic resin and embedded in plastic block molds with 100% Polybed 812. One-micron sections (thick sections) were cut on an ultra-Leica EMCU ultra microtome and placed on glass slides and stained with Toluidine Blue. Ultra-thin Sects. (80 nm) were cut from sample blocks using a Leica EMUC Ultra microtome and mounted on 100 mesh copper grids. Grids were stained with 2% uranyl acetate and Reynold’s lead stain. Grids were analyzed on a JEOL JEM 1250 electron microscope and images were captured on an AMTV600 digital camera.

### Statistical analysis

Statistical significance was evaluated using the two-tailed unpaired Student *t* test assuming unequal variances except as otherwise noted. Data are represented as means ± SEM. *P < 0.05, **P < 0.01, ***P < 0.005, ****P < 0.0005. ns is not significant.

## Supplementary Information

**Additional file 1: Table S1.** Primers for RT-PCR. Human and mouse RT-PCR primer sequences used for verification of expression in reproductive tract.

**Additional file 2: Figure S1.** Immunofluorescent staining with exogenous mouse/human RNF133-FLAG in HEK293 cells. The anti-FLAG antibody and the anti-calnexin antibody were used for RNF133 (red) and calnexin (green), respectively. Scale bar, 10 µm. This experiment was replicated three times, and representative images are presented.

**Additional file 3: Figure S2.** *Rnf148* KO mice generation. Domain structure, cDNA, genomic structure, strategy for generating *Rnf148* KO mice, and the genetic sequences deleted by the CRISPR/Cas9 system.

**Additional file 4: Figure S3.** *Rnf151* KO mice generation. Domain structure, cDNA, genomic structure, strategy for generating *Rnf151* KO mice, and the genetic sequences deleted by the CRISPR/Cas9 system.

**Additional file 5: Figure S4.** *Zswim2* KO mice generation. Domain structure, cDNA, genomic structure, strategy for generating *Zswim2* KO mice, and the genetic sequences deleted by the CRISPR/Cas9 system.

**Additional file 6: Table S2.** The single-guide RNAs (sgRNAs) to generate KO mice. The sequences of sgRNAs targeting mouse *Zswim2*, *Rnf133*, *Rnf148*, and *Rnf151*.

**Additional file 7: Figure S5.** *Rnf148*,* Rnf151*,* Zswim2 *single KO,* Rnf148/Rnf151* double KO, and* Rnf148/Rnf151/Zswim2* triple KO males remained fertile. Average litter sizes from natural mating for each of the indicated genotypes. Litter size was measured by the number of pups. Double HET males of littermates were used as controls (Ctrl) for DKO. Triple HET males of littermates were used as controls (Ctrl) for TKO. *n* ≥ 3 mice/genotype, and the data are expressed as the mean ± SEM. Individual data values for each replicate are provided in Additional file 18: Raw data.

**Additional file 8: Figure S6.** Sperm from *Rnf133*/*Rnf148* DKO males demonstrate kinematic and motility defects. A. Testis weights from *Rnf133*/*Rnf148* DKO and DHET. B. Average sperm number from caudal epididymis of *Rnf133*/*Rnf148* DKO and DHET males. C. Total motile sperm rate measured by CASA for *Rnf133* DHET and DKO sperm. D. Progressive motility rate measured by CASA for *Rnf133* DHET and DKO sperm. E. Sperm kinetics after 15 min (left) and 120 min (right) incubation in capacitation media for *Rnf133* DHET and DKO sperm. VAP, average path velocity; VCL, curvilinear velocity; VAP, straight-line velocity. A-E. *n* ≥ 3 mice/genotype, and the data are expressed as the mean ± SEM. A-E. Individual data values for each replicate are provided in Additional file 18: Raw data. F. Hyperactivated sperm rate classified by CASAnova after 15 min and 120 min incubation in capacitation media for *Rnf133* DHET and DKO sperm. *n* = 6 mice/genotype, and the data are expressed as the mean ± SEM. G. Single-negative (SYBR14-only and PI-only) and double-negative (no stain) controls to allow for proper gating of live (SYBR14+/PI-), dead (SYBR-/PI+), and dying (SYBR+/PI+) sperm populations as shown in Fig. 4E and Fig. S4I. H. Representative multichannel fluorescence staining of *Rnf133/148* DHET and DKO sperm incubated with membrane-permeant SYBR14 (green) and membrane-impermeant propidium iodide (PI; red) nucleic acid stains to identify live (SYBR14+/PI-) and dead (SYBR-/PI+) sperm. *n* = 4 mice/genotype. I. Results from flow cytometry-based quantification of the percentage of live (SYBR14+/PI- and SYBR+/PI+) and dead (SYBR-/PI+) *Rnf133/148* DHET and DKO sperm. SYBR+/PI+ cells mark a population of cells that were alive when removed from the animal but are rapidly undergoing death in vitro. *n* = 4 mice/genotype, and the data are expressed as the mean ± SEM.

**Additional file 9: Figure S7.** Sperm from *Rnf148* single KO males demonstrate no kinematic or motility defects. A. Testis weights from *Rnf148* single KOs and controls. B. Average sperm number from caudal epididymis of *Rnf148* single KO and control males. C. Total motile sperm rate measured by CASA for *Rnf148* HET and KO sperm. D. Progressive motility rate measured by CASA for *Rnf148* HET and KO sperm. E. Sperm kinetics after 15 min (left) and 120 min (right) incubation in capacitation medium for *Rnf148* HET and KO sperm. VAP, average path velocity; VCL, curvilinear velocity; VAP, straight-line velocity. F. Hyperactivated sperm rate classified by CASAnova after 15 min and 120 min incubation in capacitation medium for *Rnf148* HET and KO sperm. A-F. *n* = 3 mice/genotype, and the data are expressed as the mean ± SEM. Individual data values for each replicate are provided in Additional file 18: Raw data. G. Representative periodic acid-Schiff staining seminiferous tubules at stage IX from testes of *Rnf148* HET and KO male mice. This experiment was replicated with three mice per genotype.

**Additional file 10: Figure S8.** Sperm from *Rnf151* single KO males demonstrate no kinematic or motility defects. A. Testis weights from *Rnf151* single KOs and controls. B. Average sperm number from caudal epididymis of *Rnf151* single KO and control males. C. Total motile sperm rate measured by CASA for *Rnf151* HET and KO sperm. D. Progressive motility rate measured by CASA for *Rnf151* HET and KO sperm. E. Sperm kinetics after 15 min (left) and 120 min (right) incubation in capacitation medium for *Rnf151* HET and KO sperm. VAP, average path velocity; VCL, curvilinear velocity; VAP, straight-line velocity. F. Hyperactivated sperm rate classified by CASAnova after 15 min and 120 min incubation in capacitation medium for *Rnf151* HET and KO sperm. A-F. *n* = 3 mice/genotype, and the data are expressed as the mean ± SEM. Individual data values for each replicate are provided in Additional file 18: Raw data. G. Representative periodic acid-Schiff staining seminiferous tubules at stage IX from testes of *Rnf151* HET and KO male mice. This experiment was replicated with three mice per genotype.

**Additional file 11: Figure S9.** Sperm from *Zswim2* single KO males demonstrate no kinematic or motility defects. A. Testis weights from *Zswim2* single KOs and controls. B. Average sperm number from caudal epididymis of *Zswim2* single KO and control males. C. Total motile sperm rate measured by CASA for *Zswim2* HET and KO sperm. D. Progressive motility rate measured by CASA for *Zswim2* HET and KO sperm. E. Sperm kinetics after 15 min (left) and 120 min (right) incubation in capacitation medium for *Zswim2* HET and KO sperm. VAP, average path velocity; VCL, curvilinear velocity; VAP, straight-line velocity. F. Hyperactivated sperm rate classified by CASAnova after 15 min and 120 min incubation in capacitation medium for *Zswim2* HET and KO sperm. A-F. *n* ≥ 3 mice/genotype, and the data are expressed as the mean ± SEM. Individual data values for each replicate are provided in Additional file 18: Raw data. G. Representative periodic acid-Schiff staining seminiferous tubules at stage IX from testes of *Zswim2* HET and KO male mice. This experiment was replicated with three mice per genotype.

**Additional file 12: Figure S10.** Sperm from *Rnf148/Rnf151* double KO males demonstrate no kinematic or motility defects. A. Testis weights from *Rnf148/Rnf151* double KOs (DKO) and double HETs (DHET). B. Average sperm number from caudal epididymis of *Rnf148/Rnf151* DKO and control males. C. Total motile sperm rate measured by CASA for *Rnf148/Rnf151* DHET and DKO sperm. D. Progressive motility rate measured by CASA for *Rnf148/Rnf151* DHET and DKO sperm. E. Sperm kinetics after 15 min (left) and 120 min (right) incubation in capacitation medium for *Rnf148/Rnf151* DHET and DKO sperm. VAP, average path velocity; VCL, curvilinear velocity; VAP, straight-line velocity. F. Hyperactivated sperm rate classified by CASAnova after 15 min and 120 min incubation in capacitation medium for *Rnf148/Rnf151* DHET and DKO sperm. A-F. *n* ≥ 3 mice/genotype, and the data are expressed as the mean ± SEM. Individual data values for each replicate are provided in Additional file 18: Raw data.

**Additional file 13: Figure S11.** Western blot analysis of immunoprecipitation with exogenous mouse/human RNF133-FLAG and HA-UBE2J1. Uncropped images of captured images as shown in Fig. 6A. The anti-HA antibody was used for immunoprecipitation and the anti-FLAG antibody was used for Western blot analysis. This experiment was replicated three times, and representative blots are presented.

**Additional file 14: Figure S12.** Western blot analysis of immunoprecipitation with exogenous human RNF133-FLAG, RNF151-FLAG, HA-UBE2C, and HA-UBE2J1. Uncropped images of captured images as shown in Fig. 6A. The anti-HA antibody was used for immunoprecipitation and the anti-FLAG antibody was used for Western blot analysis. This experiment was replicated three times, and representative blots are presented.

**Additional file 15: Figure S13.** Western blot analysis of immunoprecipitation with exogenous human RNF133-mutant-FLAG and HA-UBE2J1. Uncropped images of captured images as shown in Fig. 6A. The anti-HA antibody was used for immunoprecipitation and the anti-FLAG antibody was used for Western blot analysis. This experiment was replicated three times, and representative blots are presented.

**Additional file 16: Table S3.** Primers for genotyping. The sequences of primers for genotyping wild-type or mutant alleles of HET/KO mouse lines.

**Additional file 17: Table S4.** Primers for cloning. The sequences of primers for cloning human *UBE2J1*, human *RNF133*, human *UBE2C*, human *RNF151*, mouse *Ube2J1*, mouse *Rnf133,* and human* RNF133*-mutant.

**Additional file 18.** Raw data. This file contains raw data with individual data of replicates for Figs. 2A, 3A-B, 4C, 4E, S5, S6, S7, S8, S9, and S10 (i.e., those experiments in which n < 6).

## Data Availability

All data generated or analyzed during this study are included in this published article, its supplementary information files, and publicly available repositories. All mice presented here are available from the corresponding author upon request. Raw data for Figs. 2A, 3A,B, 4C, 4E, S5, S6, S7, S8, S9, and S10 (i.e., those experiments in which *n* < 6) can be found in Additional file 18.
