# Supplementary figures and images for: The testis-specific E3 ubiquitin ligase RNF133 is required for fecundity in mice
Source: BMC Biol. 2022 Jul 13;20:161. doi: 10.1186/s12915-022-01368-2 (PMC9277888; doi:10.1186/s12915-022-01368-2)

**Fig. S1**

hRNF133-FLAG

Calnexin

Merge

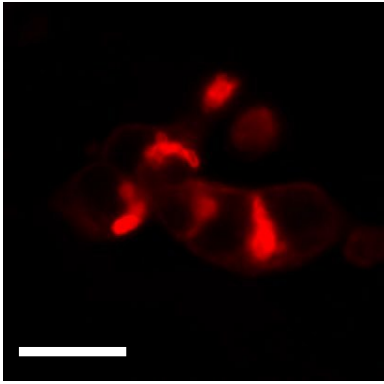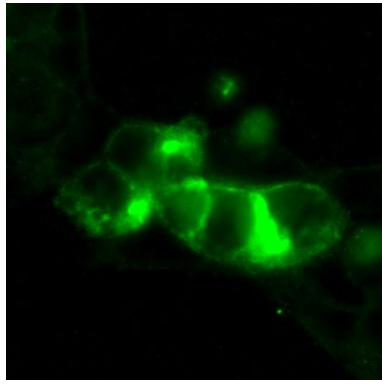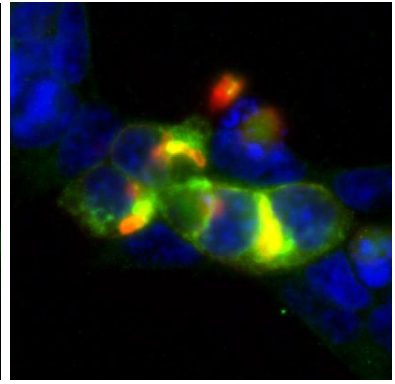

Supplement: Supplementary file 2 — Additional file 2: Figure S1. Immunofluorescent staining with exogenous mouse/human RNF133-FLAG in HEK293 cells. The anti-FLAG antibody and the anti-calnexin antibody were used for RNF133 (red) and calnexin (green), respectively. Scale bar, 10 µm. This experiment was replicated three times, and representative images are presented. [file 12915_2022_1368_MOESM2_ESM.pdf]

**Fig. S2**

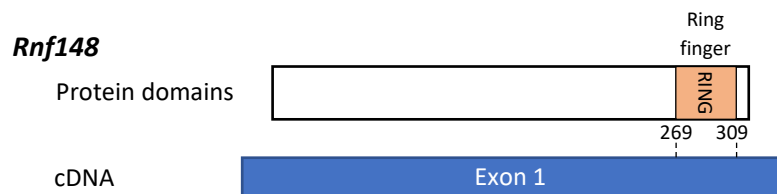

**CRISPR KO Strategy**

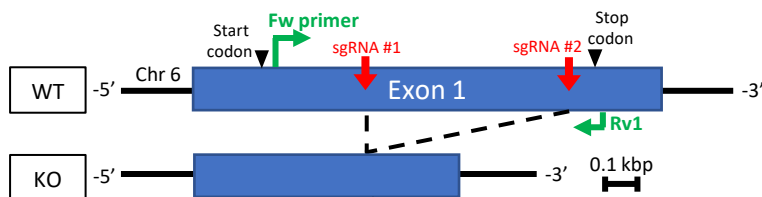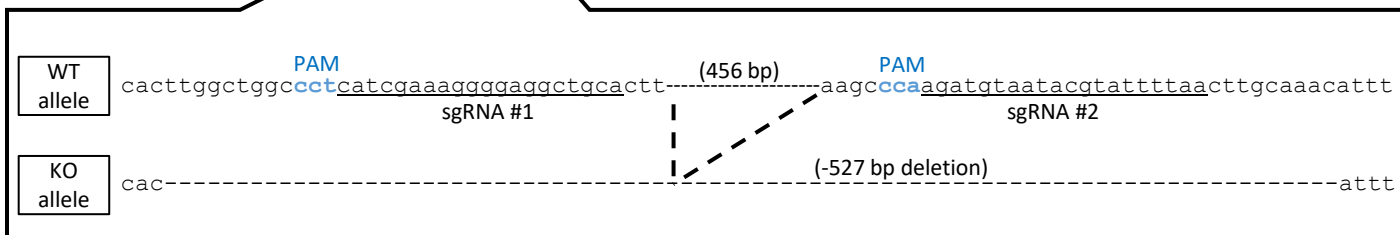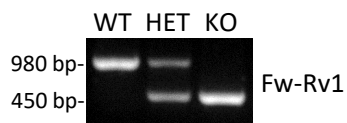

Supplement: Supplementary file 3 — Additional file 3: Figure S2. Rnf148 KO mice generation. Domain structure, cDNA, genomic structure, strategy for generating Rnf148 KO mice, and the genetic sequences deleted by the CRISPR/Cas9 system. [file 12915_2022_1368_MOESM3_ESM.pdf]

**Fig. S3**

***Rnf151***

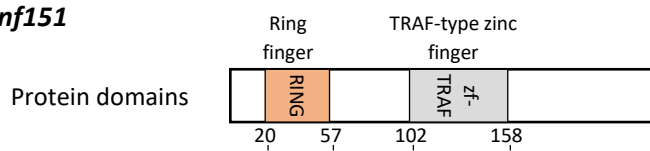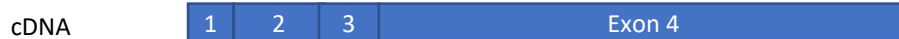

**CRISPR KO Strategy**

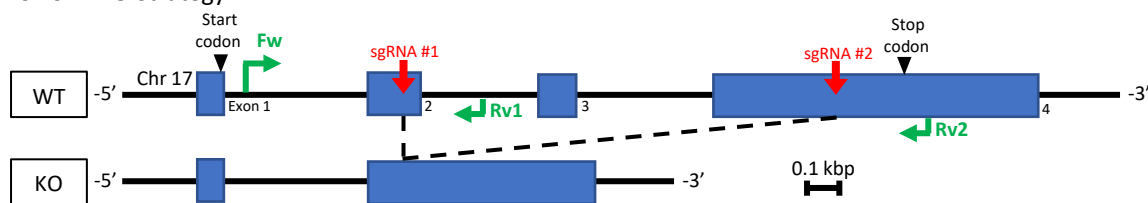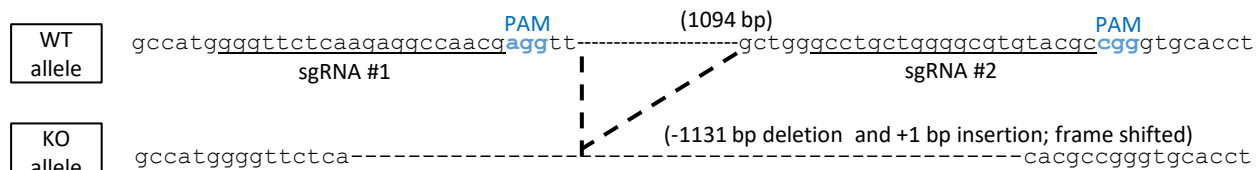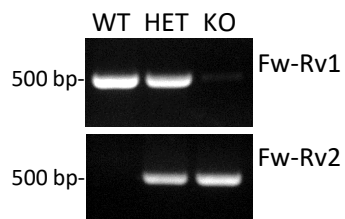

Supplement: Supplementary file 4 — Additional file 4: Figure S3. Rnf151 KO mice generation. Domain structure, cDNA, genomic structure, strategy for generating Rnf151 KO mice, and the genetic sequences deleted by the CRISPR/Cas9 system. [file 12915_2022_1368_MOESM4_ESM.pdf]

**Fig. S4**

**Zswim2**

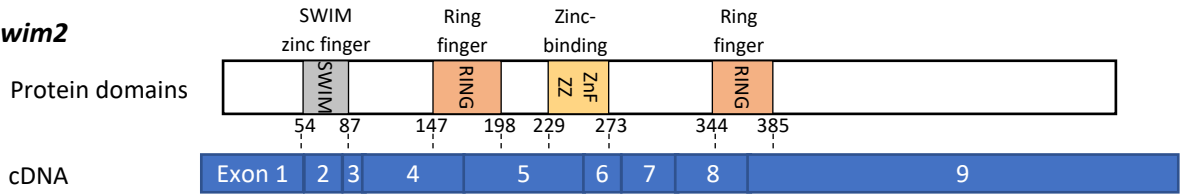

**CRISPR KO Strategy**

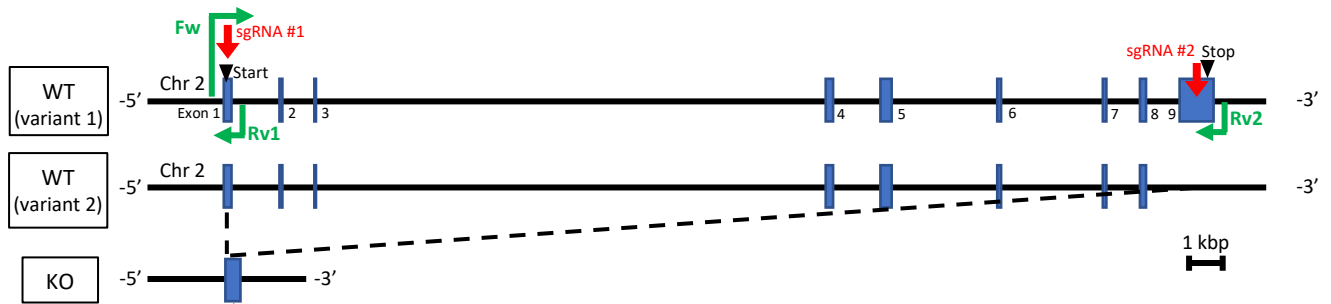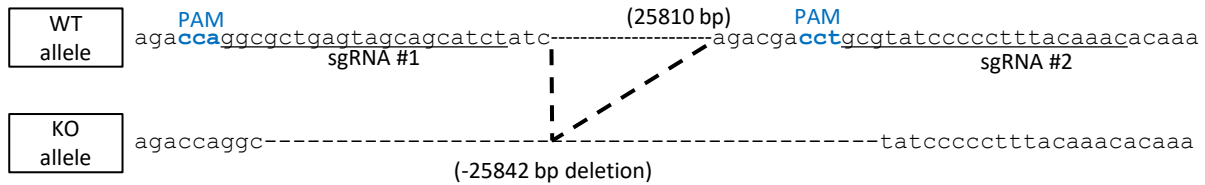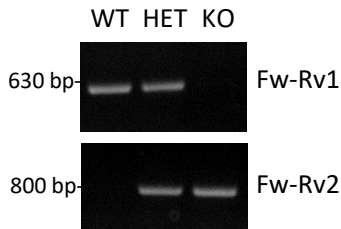

Supplement: Supplementary file 5 — Additional file 5: Figure S4. Zswim2 KO mice generation. Domain structure, cDNA, genomic structure, strategy for generating Zswim2 KO mice, and the genetic sequences deleted by the CRISPR/Cas9 system. [file 12915_2022_1368_MOESM5_ESM.pdf]

Fig. S5.

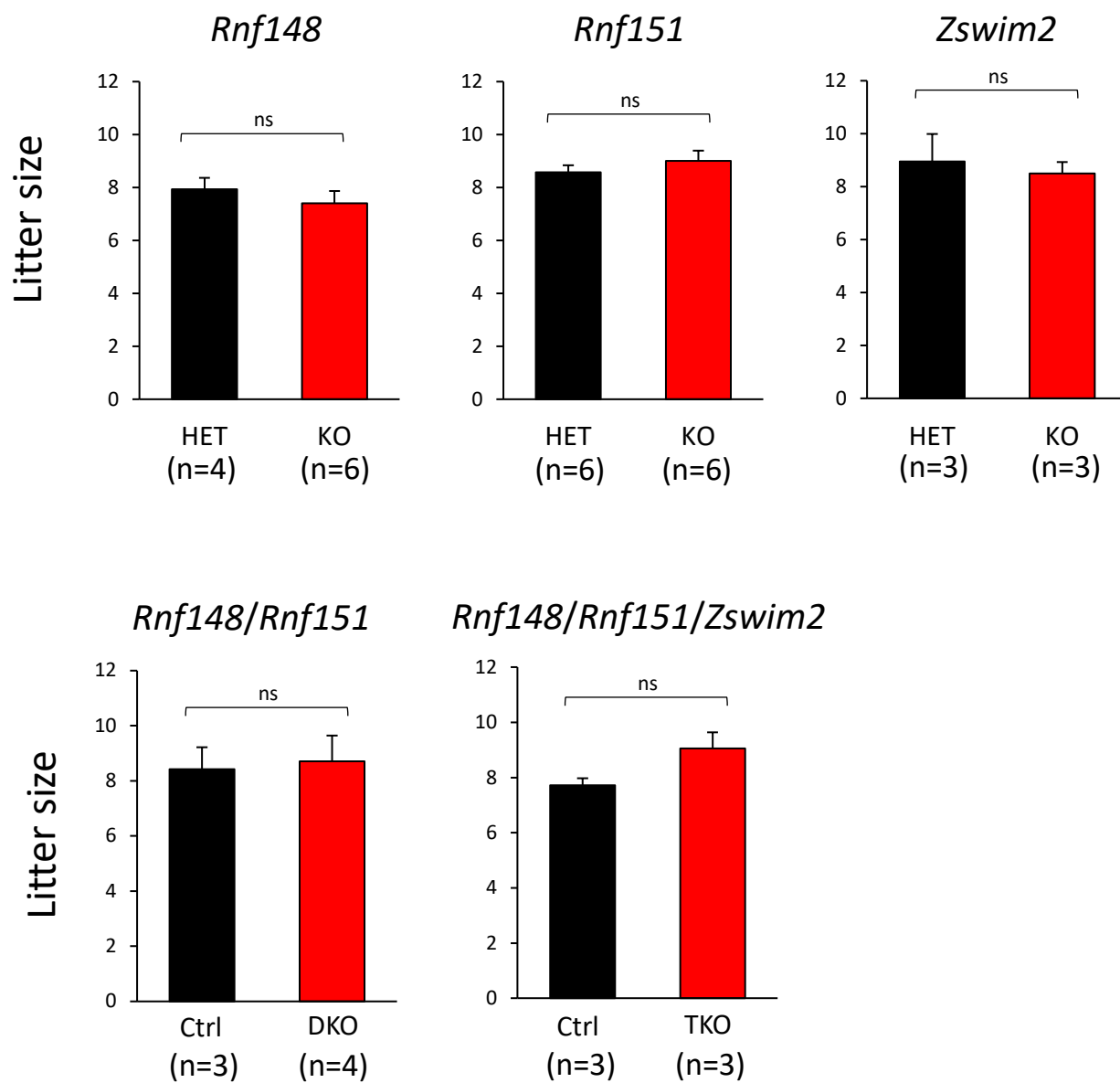

Supplement: Supplementary file 7 — Additional file 7: Figure S5. Rnf148, Rnf151, Zswim2 single KO, Rnf148/Rnf151 double KO, and Rnf148/Rnf151/Zswim2 triple KO males remained fertile. Average litter sizes from natural mating for each of the indicated genotypes. Litter size was measured by the number of pups. Double HET males of littermates were used as controls (Ctrl) for DKO. Triple HET males of littermates were used as controls (Ctrl) for TKO. n ≥ 3 mice/genotype, and the data are expressed as the mean ± SEM. Individual data values for each replicate are provided in Additional file 18: Raw data. [file 12915_2022_1368_MOESM7_ESM.pdf]

**Fig. S6. *Rnf133-Rnf148* DKO males show subfertility**

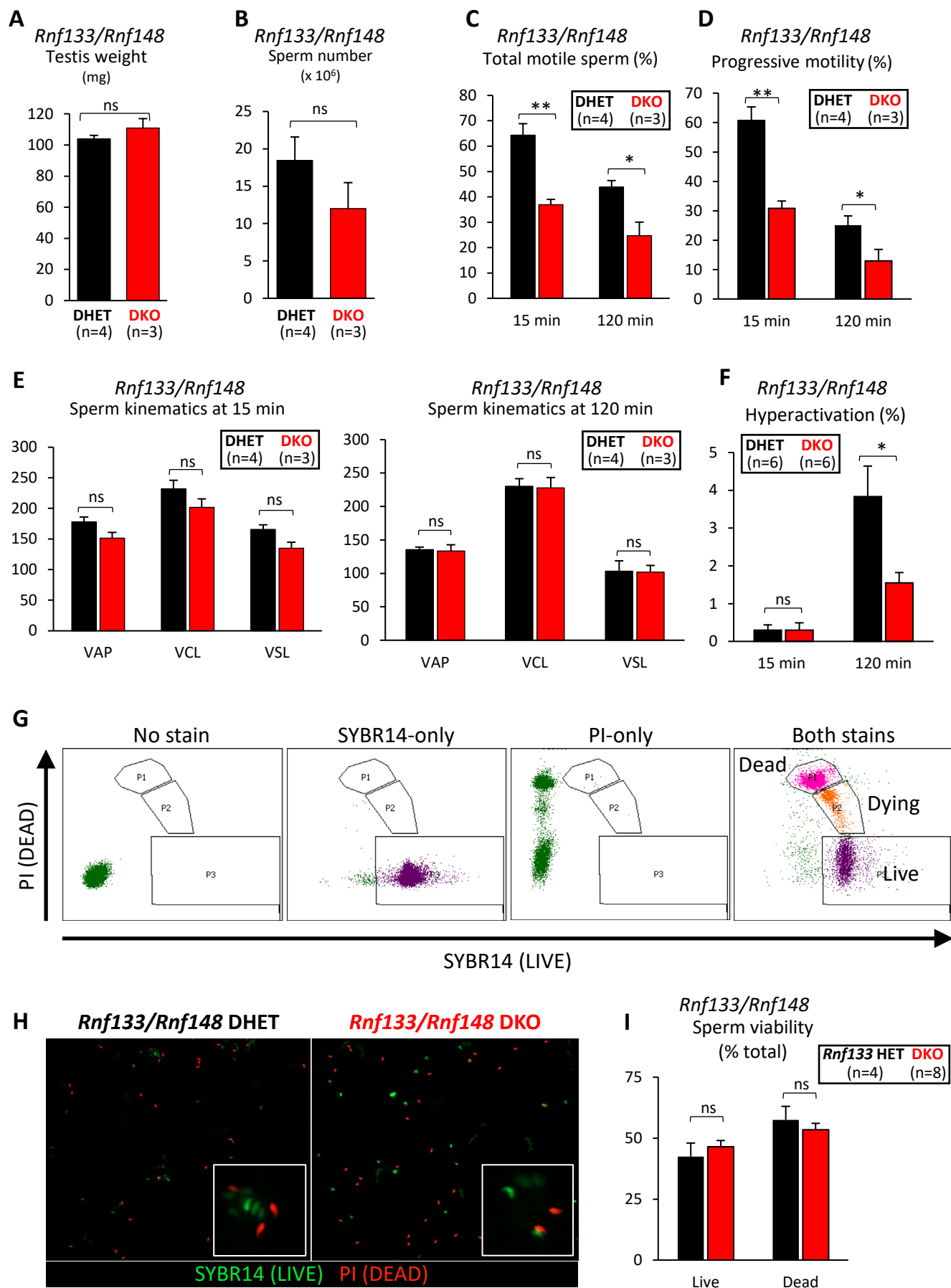

Supplement: Supplementary file 8 — Additional file 8: Figure S6. Sperm from Rnf133/Rnf148 DKO males demonstrate kinematic and motility defects. A. Testis weights from Rnf133/Rnf148 DKO and DHET. B. Average sperm number from caudal epididymis of Rnf133/Rnf148 DKO and DHET males. C. Total motile sperm rate measured by CASA for Rnf133 DHET and DKO sperm. D. Progressive motility rate measured by CASA for Rnf133 DHET and DKO sperm. E. Sperm kinetics after 15 min (left) and 120 min (right) incubation in capacitation media for Rnf133 DHET and DKO sperm. VAP, average path velocity; VCL, curvilinear velocity; VAP, straight-line velocity. A-E. n ≥ 3 mice/genotype, and the data are expressed as the mean ± SEM. A-E. Individual data values for each replicate are provided in Additional file 18: Raw data. F. Hyperactivated sperm rate classified by CASAnova after 15 min and 120 min incubation in capacitation media for Rnf133 DHET and DKO sperm. n = 6 mice/genotype, and the data are expressed as the mean ± SEM. G. Single-negative (SYBR14-only and PI-only) and double-negative (no stain) controls to allow for proper gating of live (SYBR14+/PI-), dead (SYBR-/PI+), and dying (SYBR+/PI+) sperm populations as shown in Fig. 4E and Fig. S4I. H. Representative multichannel fluorescence staining of Rnf133/148 DHET and DKO sperm incubated with membrane-permeant SYBR14 (green) and membrane-impermeant propidium iodide (PI; red) nucleic acid stains to identify live (SYBR14+/PI-) and dead (SYBR-/PI+) sperm. n = 4 mice/genotype. I. Results from flow cytometry-based quantification of the percentage of live (SYBR14+/PI- and SYBR+/PI+) and dead (SYBR-/PI+) Rnf133/148 DHET and DKO sperm. SYBR+/PI+ cells mark a population of cells that were alive when removed from the animal but are rapidly undergoing death in vitro. n = 4 mice/genotype, and the data are expressed as the mean ± SEM. [file 12915_2022_1368_MOESM8_ESM.pdf]

**Fig. S7. *Rnf148* KO testis & sperm analysis**

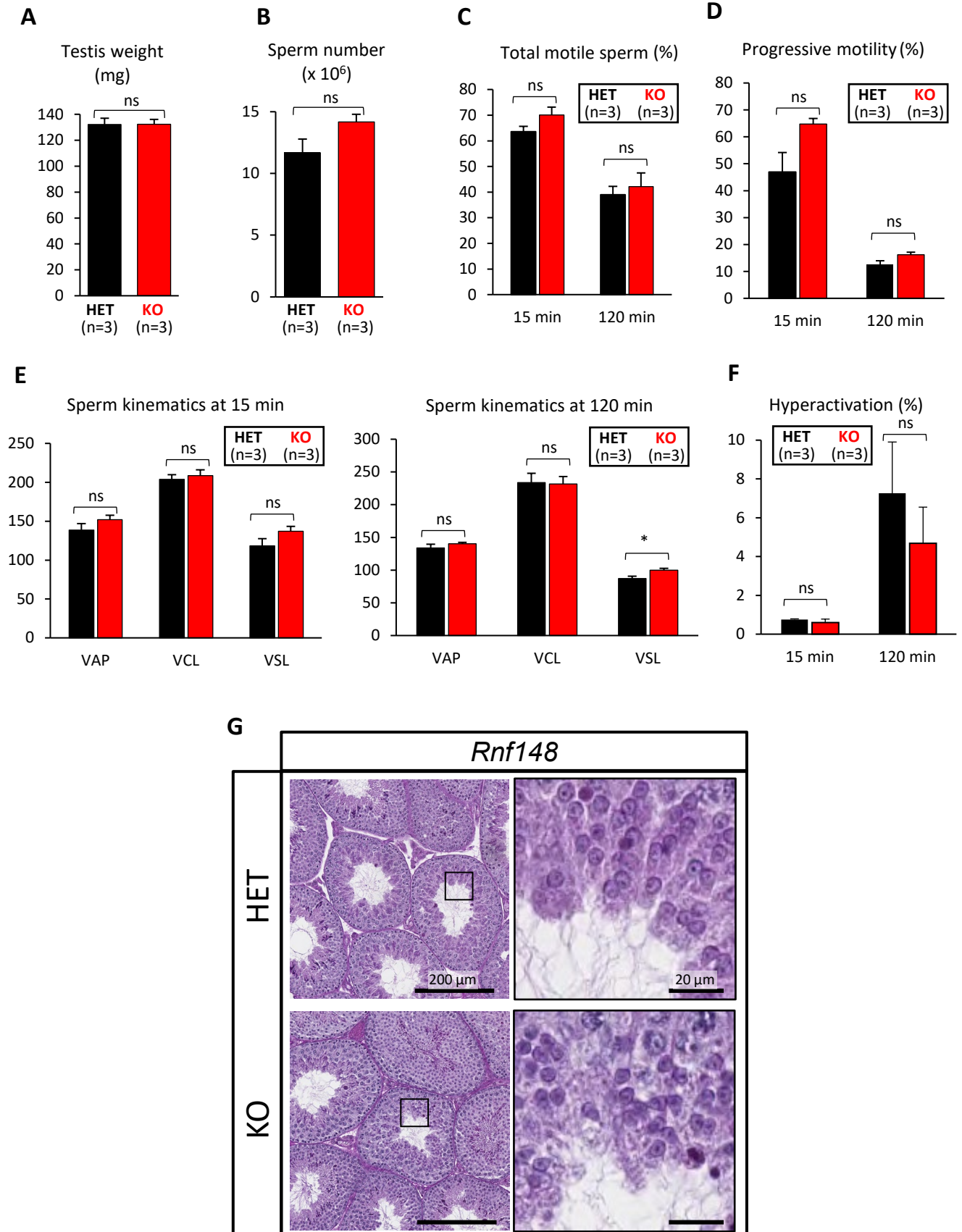

Supplement: Supplementary file 9 — Additional file 9: Figure S7. Sperm from Rnf148 single KO males demonstrate no kinematic or motility defects. A. Testis weights from Rnf148 single KOs and controls. B. Average sperm number from caudal epididymis of Rnf148 single KO and control males. C. Total motile sperm rate measured by CASA for Rnf148 HET and KO sperm. D. Progressive motility rate measured by CASA for Rnf148 HET and KO sperm. E. Sperm kinetics after 15 min (left) and 120 min (right) incubation in capacitation medium for Rnf148 HET and KO sperm. VAP, average path velocity; VCL, curvilinear velocity; VAP, straight-line velocity. F. Hyperactivated sperm rate classified by CASAnova after 15 min and 120 min incubation in capacitation medium for Rnf148 HET and KO sperm. A-F. n = 3 mice/genotype, and the data are expressed as the mean ± SEM. Individual data values for each replicate are provided in Additional file 18: Raw data. G. Representative periodic acid-Schiff staining seminiferous tubules at stage IX from testes of Rnf148 HET and KO male mice. This experiment was replicated with three mice per genotype. [file 12915_2022_1368_MOESM9_ESM.pdf]

**Fig. S8. *Rnf151* KO testis & sperm analysis**

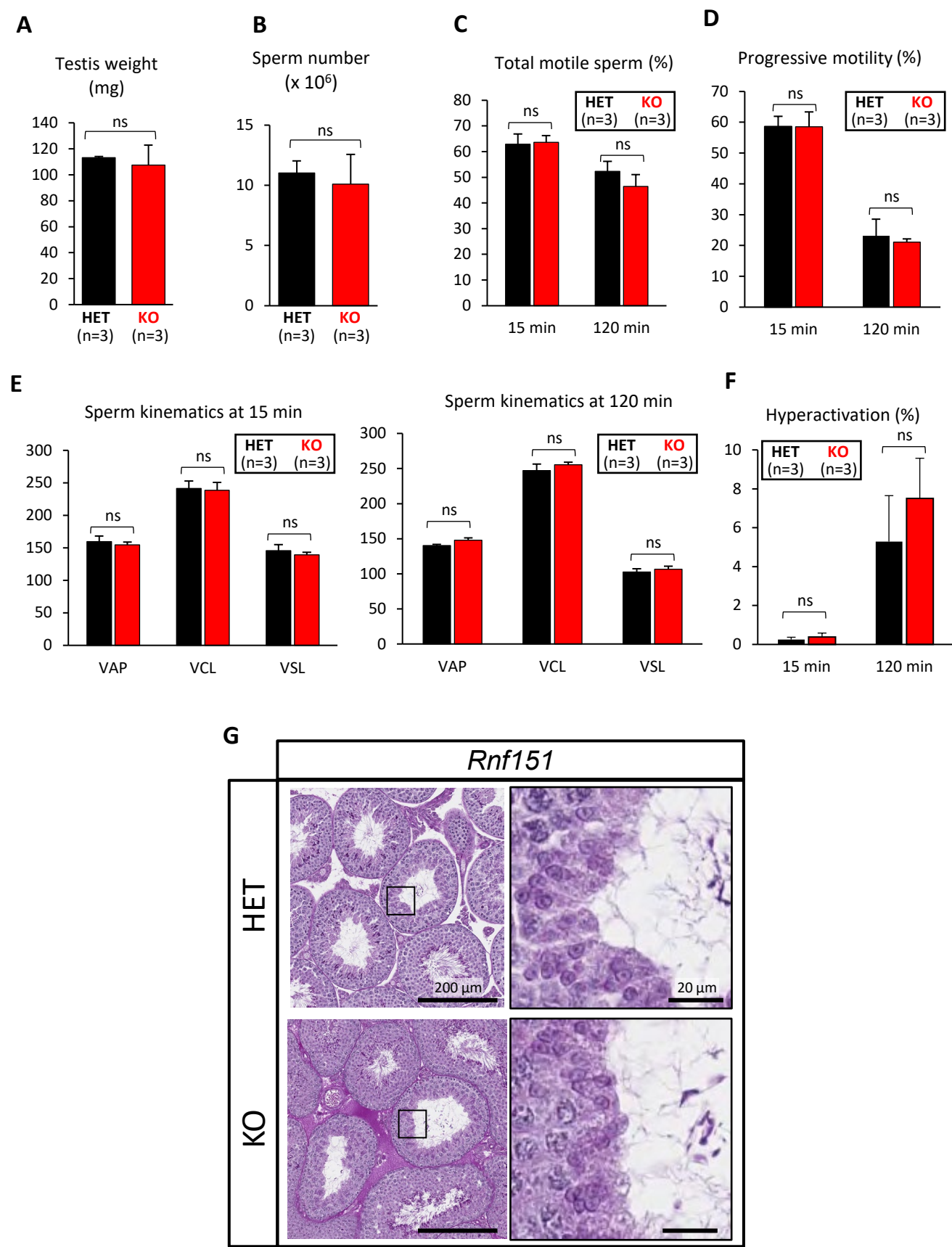

Supplement: Supplementary file 10 — Additional file 10: Figure S8. Sperm from Rnf151 single KO males demonstrate no kinematic or motility defects. A. Testis weights from Rnf151 single KOs and controls. B. Average sperm number from caudal epididymis of Rnf151 single KO and control males. C. Total motile sperm rate measured by CASA for Rnf151 HET and KO sperm. D. Progressive motility rate measured by CASA for Rnf151 HET and KO sperm. E. Sperm kinetics after 15 min (left) and 120 min (right) incubation in capacitation medium for Rnf151 HET and KO sperm. VAP, average path velocity; VCL, curvilinear velocity; VAP, straight-line velocity. F. Hyperactivated sperm rate classified by CASAnova after 15 min and 120 min incubation in capacitation medium for Rnf151 HET and KO sperm. A-F. n = 3 mice/genotype, and the data are expressed as the mean ± SEM. Individual data values for each replicate are provided in Additional file 18: Raw data. G. Representative periodic acid-Schiff staining seminiferous tubules at stage IX from testes of Rnf151 HET and KO male mice. This experiment was replicated with three mice per genotype. [file 12915_2022_1368_MOESM10_ESM.pdf]

**Fig. S9. *Zswim2* KO testis & sperm analysis**

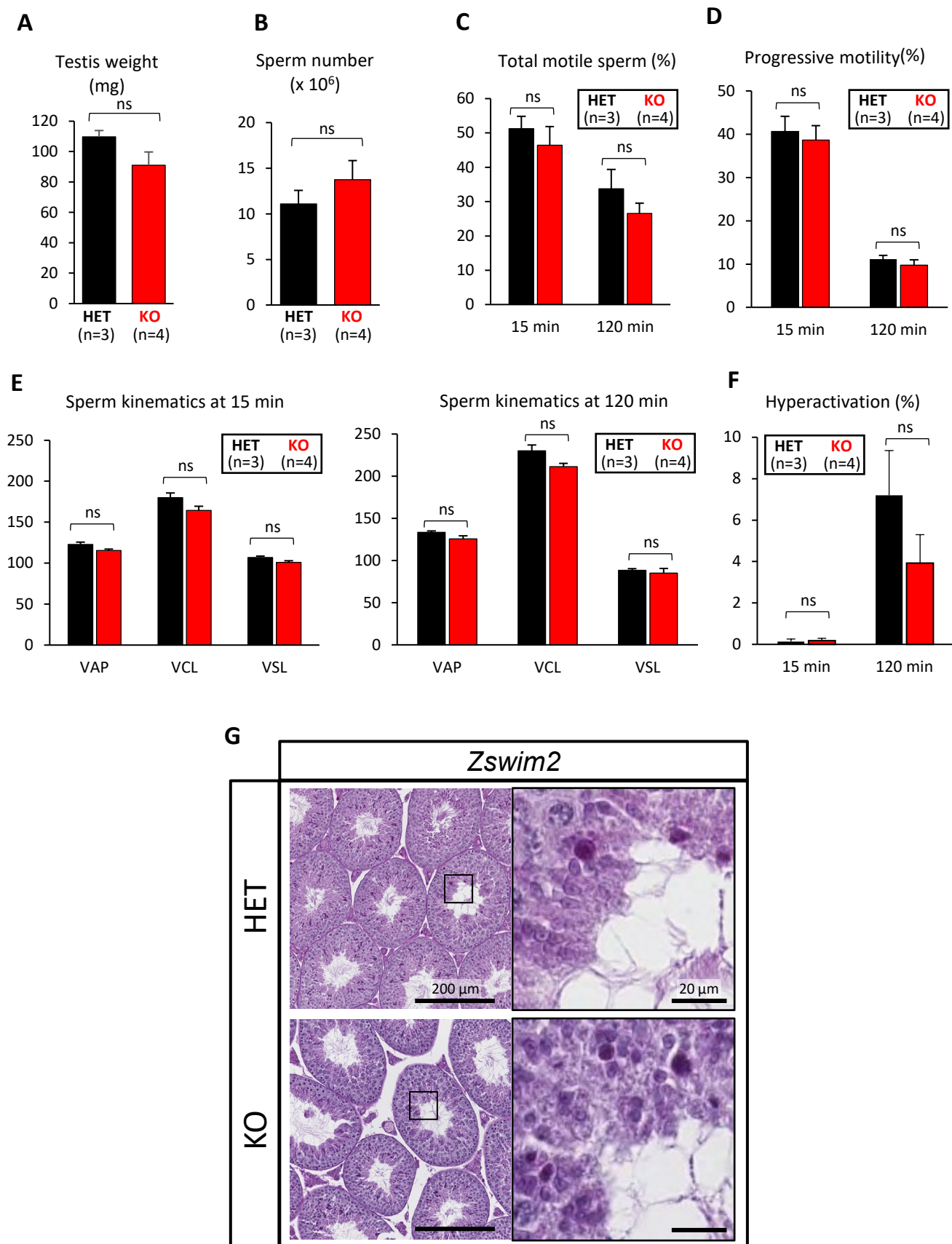

Supplement: Supplementary file 11 — Additional file 11: Figure S9. Sperm from Zswim2 single KO males demonstrate no kinematic or motility defects. A. Testis weights from Zswim2 single KOs and controls. B. Average sperm number from caudal epididymis of Zswim2 single KO and control males. C. Total motile sperm rate measured by CASA for Zswim2 HET and KO sperm. D. Progressive motility rate measured by CASA for Zswim2 HET and KO sperm. E. Sperm kinetics after 15 min (left) and 120 min (right) incubation in capacitation medium for Zswim2 HET and KO sperm. VAP, average path velocity; VCL, curvilinear velocity; VAP, straight-line velocity. F. Hyperactivated sperm rate classified by CASAnova after 15 min and 120 min incubation in capacitation medium for Zswim2 HET and KO sperm. A-F. n ≥ 3 mice/genotype, and the data are expressed as the mean ± SEM. Individual data values for each replicate are provided in Additional file 18: Raw data. G. Representative periodic acid-Schiff staining seminiferous tubules at stage IX from testes of Zswim2 HET and KO male mice. This experiment was replicated with three mice per genotype. [file 12915_2022_1368_MOESM11_ESM.pdf]

**Fig. S10 *Rnf148/Rnf151* DKO testis & sperm analysis**

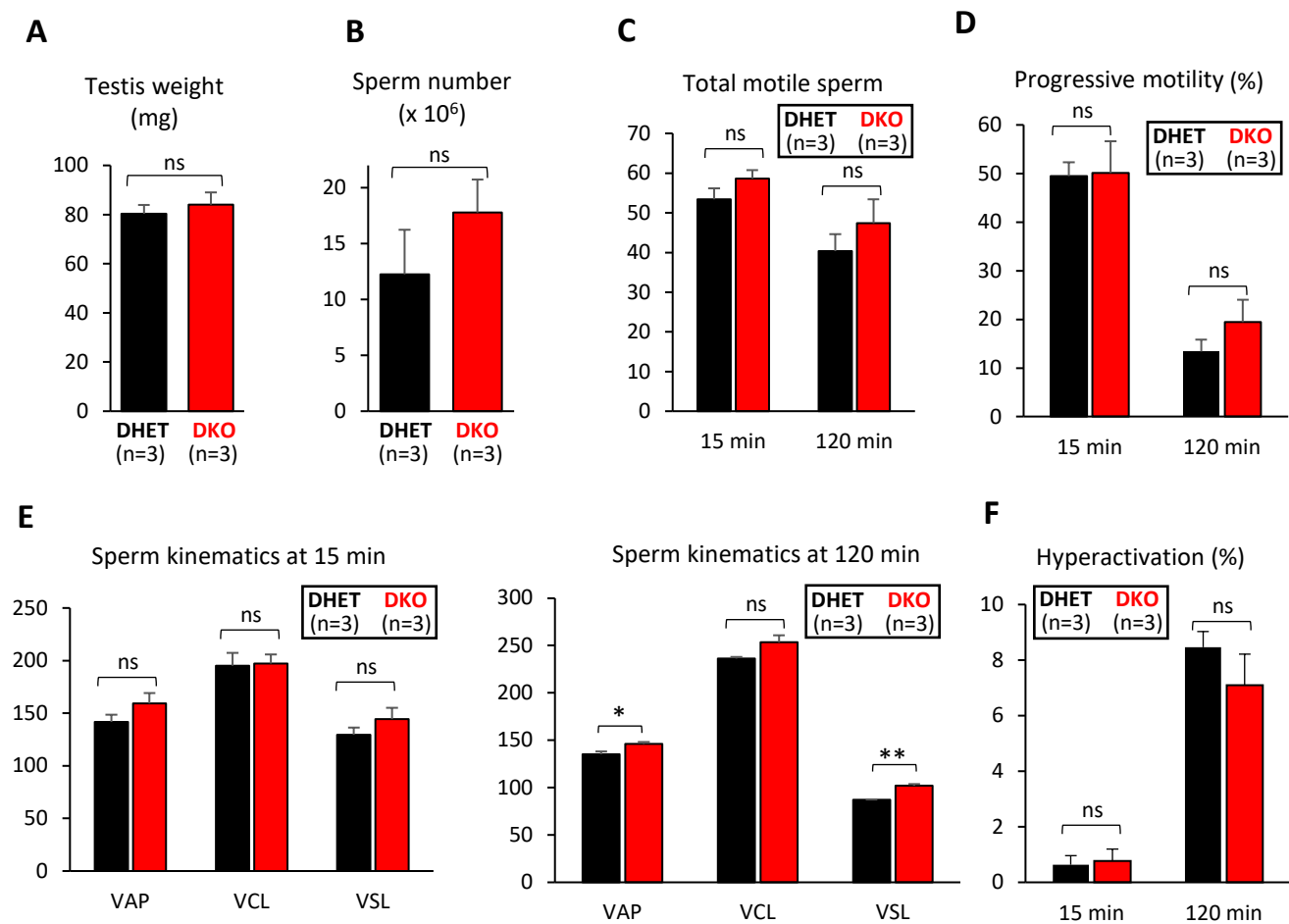

Supplement: Supplementary file 12 — Additional file 12: Figure S10. Sperm from Rnf148/Rnf151 double KO males demonstrate no kinematic or motility defects. A. Testis weights from Rnf148/Rnf151 double KOs (DKO) and double HETs (DHET). B. Average sperm number from caudal epididymis of Rnf148/Rnf151 DKO and control males. C. Total motile sperm rate measured by CASA for Rnf148/Rnf151 DHET and DKO sperm. D. Progressive motility rate measured by CASA for Rnf148/Rnf151 DHET and DKO sperm. E. Sperm kinetics after 15 min (left) and 120 min (right) incubation in capacitation medium for Rnf148/Rnf151 DHET and DKO sperm. VAP, average path velocity; VCL, curvilinear velocity; VAP, straight-line velocity. F. Hyperactivated sperm rate classified by CASAnova after 15 min and 120 min incubation in capacitation medium for Rnf148/Rnf151 DHET and DKO sperm. A-F. n ≥ 3 mice/genotype, and the data are expressed as the mean ± SEM. Individual data values for each replicate are provided in Additional file 18: Raw data. [file 12915_2022_1368_MOESM12_ESM.pdf]

**Fig. S11**

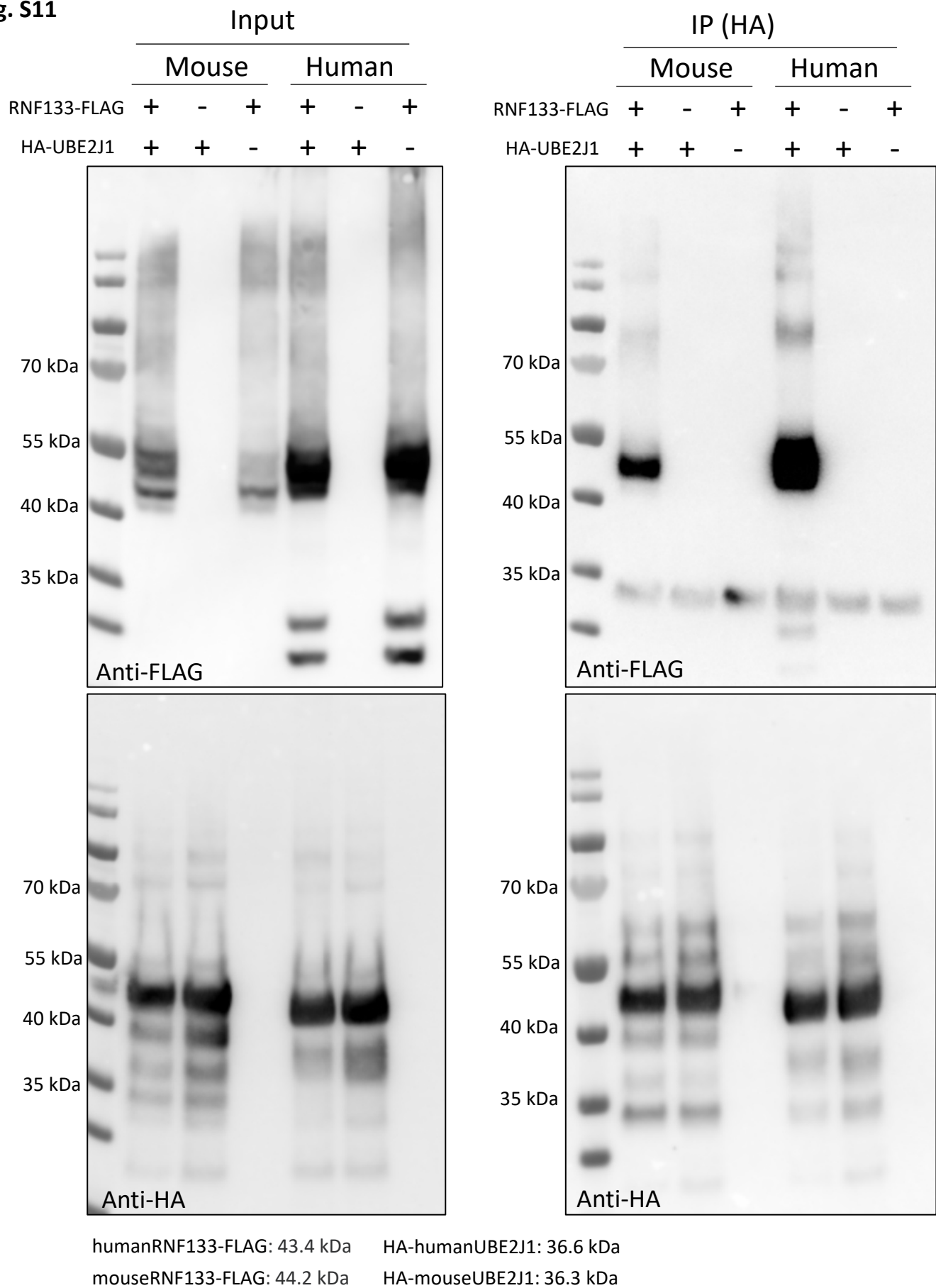

Supplement: Supplementary file 13 — Additional file 13: Figure S11. Western blot analysis of immunoprecipitation with exogenous mouse/human RNF133-FLAG and HA-UBE2J1. Uncropped images of captured images as shown in Fig. 6A. The anti-HA antibody was used for immunoprecipitation and the anti-FLAG antibody was used for Western blot analysis. This experiment was replicated three times, and representative blots are presented. [file 12915_2022_1368_MOESM13_ESM.pdf]

Fig. S13

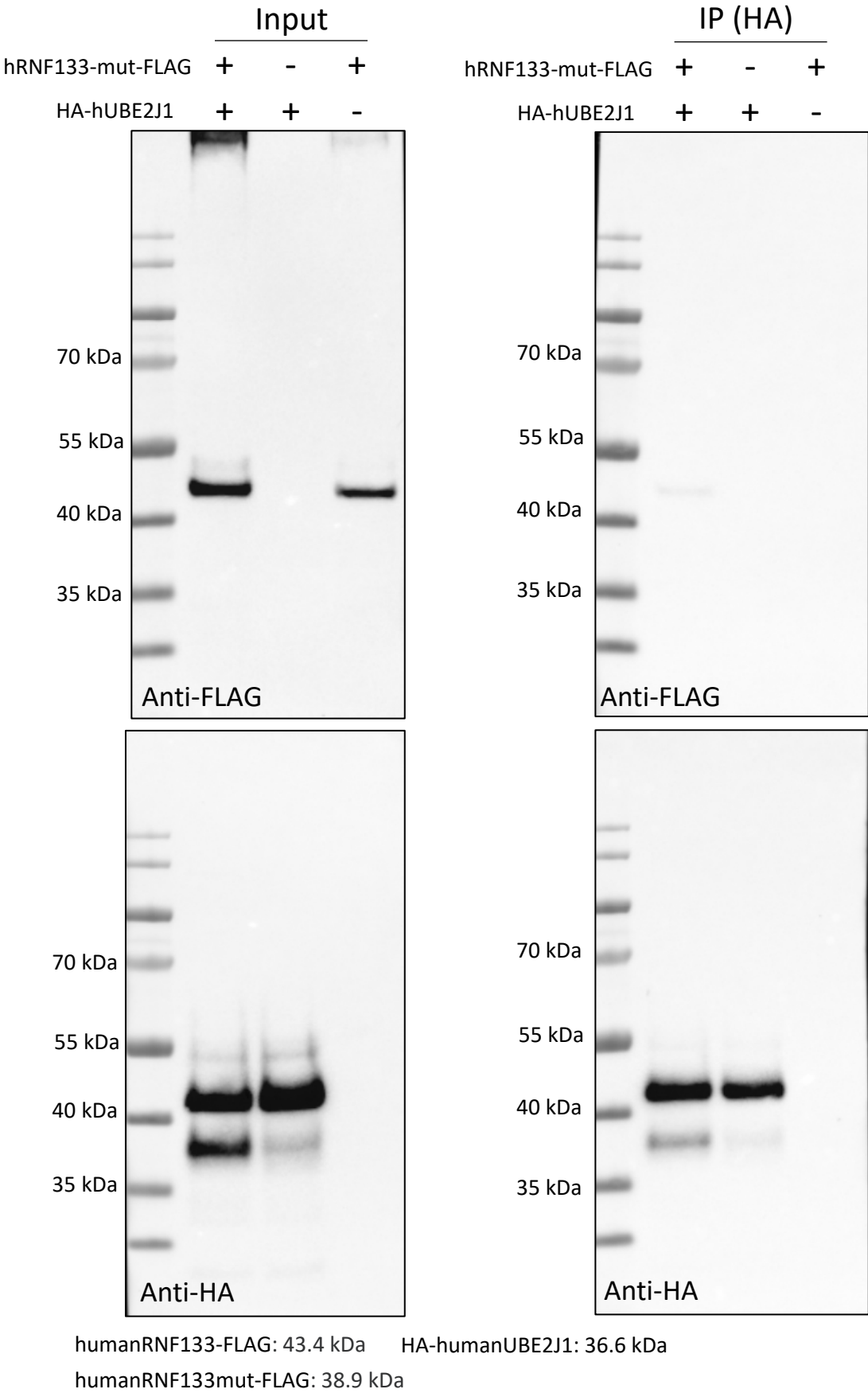

Supplement: Supplementary file 15 — Additional file 15: Figure S13. Western blot analysis of immunoprecipitation with exogenous human RNF133-mutant-FLAG and HA-UBE2J1. Uncropped images of captured images as shown in Fig. 6A. The anti-HA antibody was used for immunoprecipitation and the anti-FLAG antibody was used for Western blot analysis. This experiment was replicated three times, and representative blots are presented. [file 12915_2022_1368_MOESM15_ESM.pdf]
