# Supplementary material for: The testis-specific E3 ubiquitin ligase RNF133 is required for fecundity in mice
Source: BMC Biol. 2022 Jul 13;20:161. doi: 10.1186/s12915-022-01368-2 (PMC9277888; doi:10.1186/s12915-022-01368-2)
Supplement: Supplementary file 14 — Additional file 14: Figure S12. Western blot analysis of immunoprecipitation with exogenous human RNF133-FLAG, RNF151-FLAG, HA-UBE2C, and HA-UBE2J1. Uncropped images of captured images as shown in Fig. 6A. The anti-HA antibody was used for immunoprecipitation and the anti-FLAG antibody was used for Western blot analysis. This experiment was replicated three times, and representative blots are presented. [file 12915_2022_1368_MOESM14_ESM.pdf]

Fig. S12

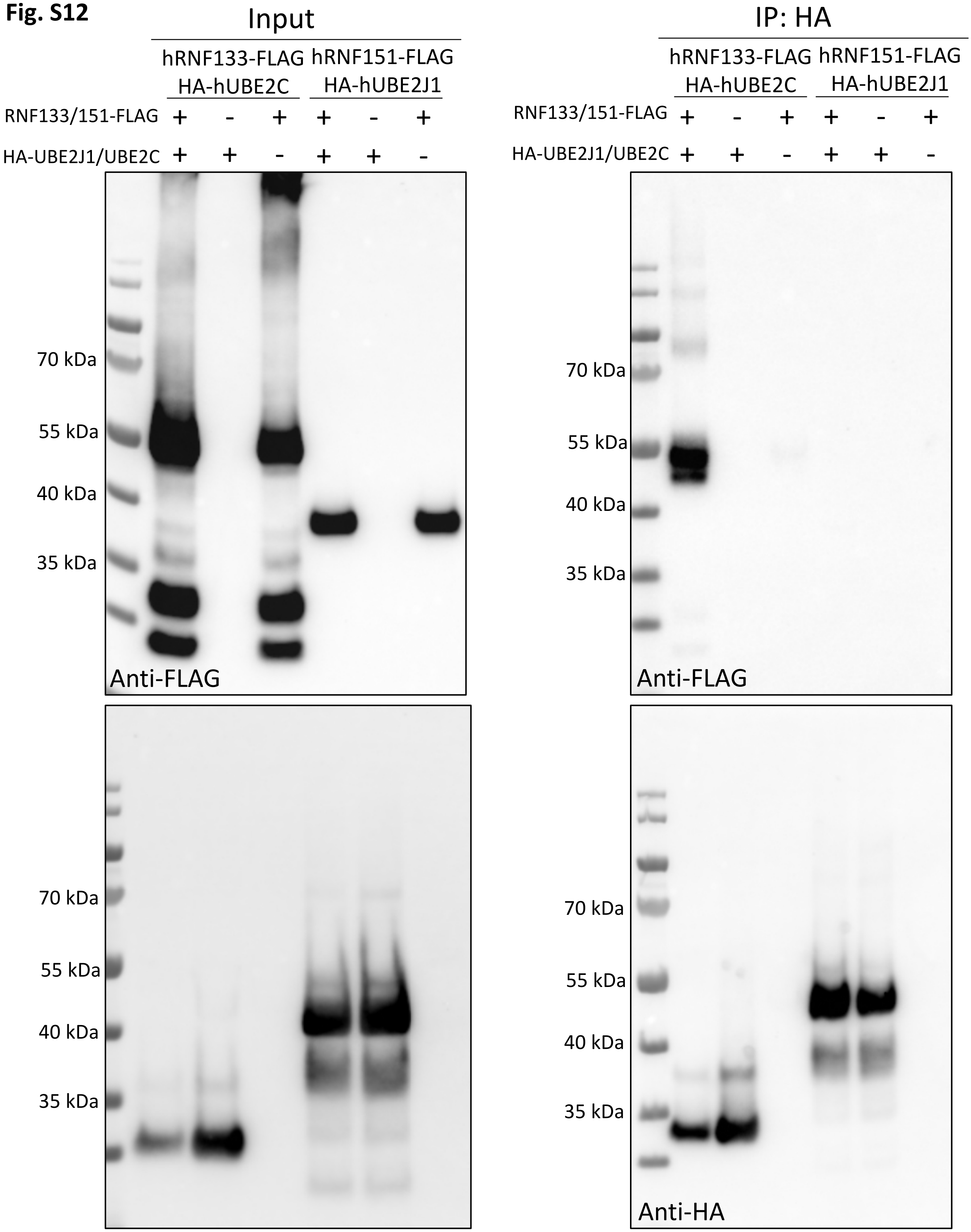

humanRNF133-FLAG: 43.4 kDa
humanRNF151-FLAG: 28.6 kDa
HA-humanUBE2J1: 36.6 kDa
HA-humanUBE2C: 21.0 kDa
